# Solvent Tuning Excited State Structural Dynamics in a Novel Bianthryl

**DOI:** 10.1021/acs.jpclett.2c03469

**Published:** 2023-01-03

**Authors:** Palas Roy, Faisal Al-Kahtani, Andrew N. Cammidge, Stephen R. Meech

**Affiliations:** School of Chemistry, University of East Anglia, Norwich NR4 7TJ, U.K.

## Abstract

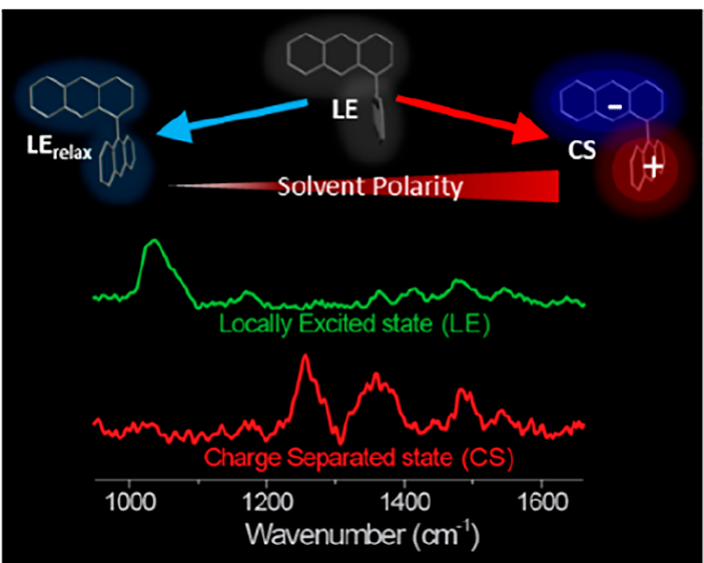

Symmetry breaking charge separation (SBCS) is central to photochemical energy conversion. The widely studied 9,9-bianthryl (9,9′BA) is the prototype, but the role of bianthryl structure is hardly investigated. Here we investigate excited state structural dynamics in a bianthryl of reduced symmetry, 1,9-bianthryl (1,9′BA), through ultrafast electronic and vibrational spectroscopy. Resonance selective Raman in polar solvents reveals a Franck–Condon state mode that disappears concomitant with the rise of ring breathing modes of radical species. Solvent-dependent dynamics show that CS is driven by solvent orientational motion, as in 9,9′BA. In nonpolar solvents the excited state undergoes multistep structural relaxation, including subpicosecond Franck–Condon state decay and biexponential diffusion-controlled structural evolution to a distorted slightly polar state. These data suggest two possible routes to SBCS; the established solvent driven pathway in rapidly relaxing polar solvents and, in slowly relaxing media, initial intramolecular reorganization to a polar structure which drives solvent orientational relaxation.

Bianthryls (BAs) played a key role in the development of photophysics.^1^ In particular, 9,9′BA is the prototype for symmetry breaking charge separation (SBCS), a topic which continues to excite wide interest.^2−4^ Lippert, Mataga, and co-workers showed that this symmetrical (*D*_2*d*_) molecule unexpectedly supports charge transfer type emission in polar solvents.^5−8^ This anomaly was widely investigated, and in an important series of papers Barbara and co-workers showed that the dynamics of CS in 9,9′BA closely follow those of solvation, suggesting a dominant role for solvent orientation dynamics in symmetry breaking and CS state stabilization.^9,10^ Given the apparently dominant role of the solvent, it seems pertinent to ask whether the initial molecular symmetry plays a role in the CS. To date, there is only one photophysical study of other unsubstituted BAs;^11^ here we employ ultrafast transient absorption (TA) and femtosecond stimulated Raman spectroscopy (FSRS) to study excited state structural dynamics in the newly synthesized 1,9′BA, which has both reduced symmetry compared to 9,9′BA (*C*_*s*_) and its two anthracene moieties are linked at different carbons, giving them slightly different electronic structures.^12^

The steady state absorption and fluorescence spectra of 1,9′BA are shown in Figure 1a (solid and dashed line, respectively) as a function of solvent polarity. The absorption spectra show that the initial excitation is to a locally excited (LE) state of anthracene and independent of solvent. The well-resolved vibronic structure with a prominent 0–0 transition near 400 nm is characteristic of a substituted anthracene monomer. This suggests negligible inter-ring coupling in the electronic ground state. Density functional theory (DFT) calculations (see the Supporting Information) support this, showing that the two rings are orthogonal to one another (Figure 1b). In contrast, the emission spectra do reflect characteristics of inter-ring coupling. Even in the least polar solvents (e.g., cyclohexane, ε = 2.02) a mirror-image relationship to the structured absorption spectrum is not observed (unlike for monomeric anthracenes^13^), but instead the emission is a single weakly structured band. The lack of mirror-image emission is indicative of a structure change in the excited state of 1,9′BA even in nonpolar solvents. This is confirmed by the observation that its fluorescence spectrum in a methylcyclohexane glass at 77 K, where large-scale structural reorganization is suppressed, recovers the structured mirror-image emission (Supporting Information, Figure S1). This observation of room temperature excited state structure change is consistent with TD-DFT calculations, which show a minimum-energy structure for S_1_ with the two anthracene rings twisted at a dihedral angle of 63° and with a slight deviation from planarity in one ring (Figure 1b). This structure change is accompanied by a shortening of the inter-ring CC single bond from 1.497 to 1.469 Å and an increase in the calculated dipole moment from ≈0 D (in S_0_) to around 1 D in S_1_. A similar structure change was calculated and observed for 9,9′BA.^14−16^

**Figure 1:**
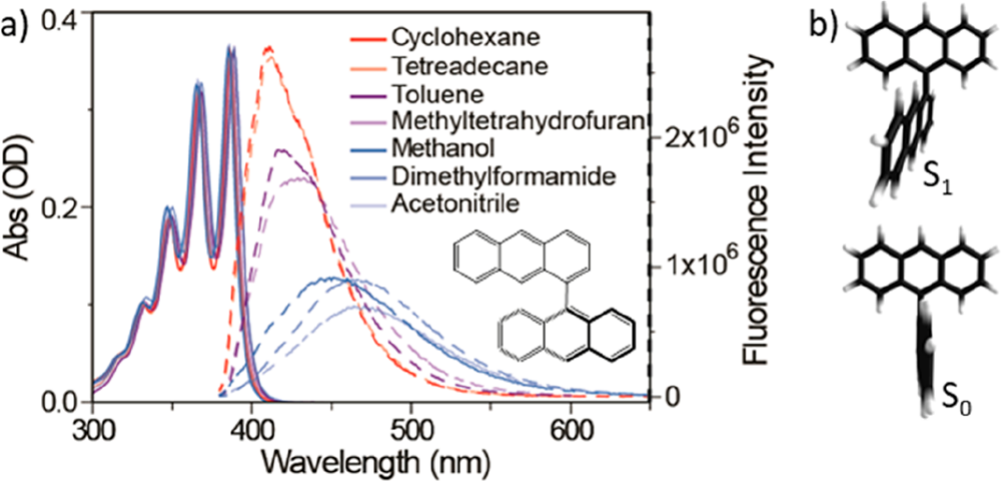
(a) Steady state absorption (solid line) and emission (broken line) spectra of 1,9′ BA in different solvents. The inset shows the chemical structure of 1,9′ BA. Emission spectra are normalized for light absorption. (b) DFT and TDDFT optimized structure of 1,9′BA in S_0_ and S_1_ states.

The emission spectrum is a strong function of solvent, shifting to the red with increasing polarity and becoming broad and unstructured. This is characteristic of CS state formation in polar media. Measurement of the solvent-polarity-dependent emission Stokes shift provides an estimate of the change in the molecular dipole moment upon excitation. The Lippert–Mataga plot relates the Stokes shift to solvent orientation polarization by  with  in which Δμ is the difference in dipole moment between the excited and ground states, *h* is Planck’s constant, *c* is the velocity of light, ρ is the Onsager radius of the molecule, ε and *n* are the solvent dielectric constant and refractive index, respectively.^17,18^ Using 5 Å as the Onsager radius for 1,9′BA (from the DFT calculated molar volume) and fitting the Stokes shifts in a range of solvents (Figure 1a) yields a dipole moment increase upon excitation of 10.4 D, suggesting CS in polar solvents. The details of the Lippert–Mataga plots are summarized in the Supporting Information (Figure S2 and Tables S1–S2). It is interesting to note that the spectral shifts for 1,9′BA are similar to 9,9′BA,^19^ and both are greater than reported for the more flexible 2,9′BA.^20^

Even in slightly polar solvents, such as toluene (ε = 2.4) and tetrahydrofuran (ε = 7.6), the peak emission intensity shifts and decreases, while in polar solvents the strongly red-shifted emission has a reduced quantum yield relative to nonpolar solvent (the fluorescence quantum yield of 1,9′BA in cyclohexane was measured as 0.54 by a relative method (see the Supporting Information)). The solvent-dependent fluorescence lifetime of 1,9′BA was also measured and observed to increase with increasing solvent polarity from 3.6 ns in cyclohexane to 12 ns in dimethylformamide (DMF) (Figure S3 and Table S3). The red-shift, attenuation, and broadening of the emission (Figure 1a) are thus accompanied by an increasing radiative lifetime, which is again characteristic of the formation of a CS state in polar solvents.

The ultrafast TA spectra of 1,9′BA in acetonitrile (ε = 37.5) are shown in Figure 2a along with the evolution associated difference spectra (EADS) recovered from a global kinetic analysis assuming a sequential model. The time-resolved spectra (Figure 2a, top panel) show a transient absorption at around 590 nm within 200 fs of photoexcitation, decreasing in amplitude on a picosecond time scale, with a corresponding increase in absorption at 680 nm. This excited state then decays on a nanosecond time scale, and at the same time a long-lived (on the nanosecond time scale) transient is formed at 450 nm. We associate the 590 nm band, which is already present at *t* = 200 fs, with the Franck–Condon (FC) LE state. The band forming at 680 nm is assigned to the CS state on the basis of its sensitivity to solvent polarity (Figure S9 showing a 10 nm red-shifted absorption between ACN and DMF) as well as previous reports of the anthracene cation radical absorption spectrum, confirmed for the electronic ground state in Figure S20.^21^ The final 450 nm transient is similar to the absorption reported for anthracene in its lowest triplet state.^22^ In addition, TA measurements in the near-IR region (Figure 2a) reveal a transient band at 980 nm which red-shifts to 1055 nm and loses oscillator strength as the CS state is formed.

**Figure 2:**
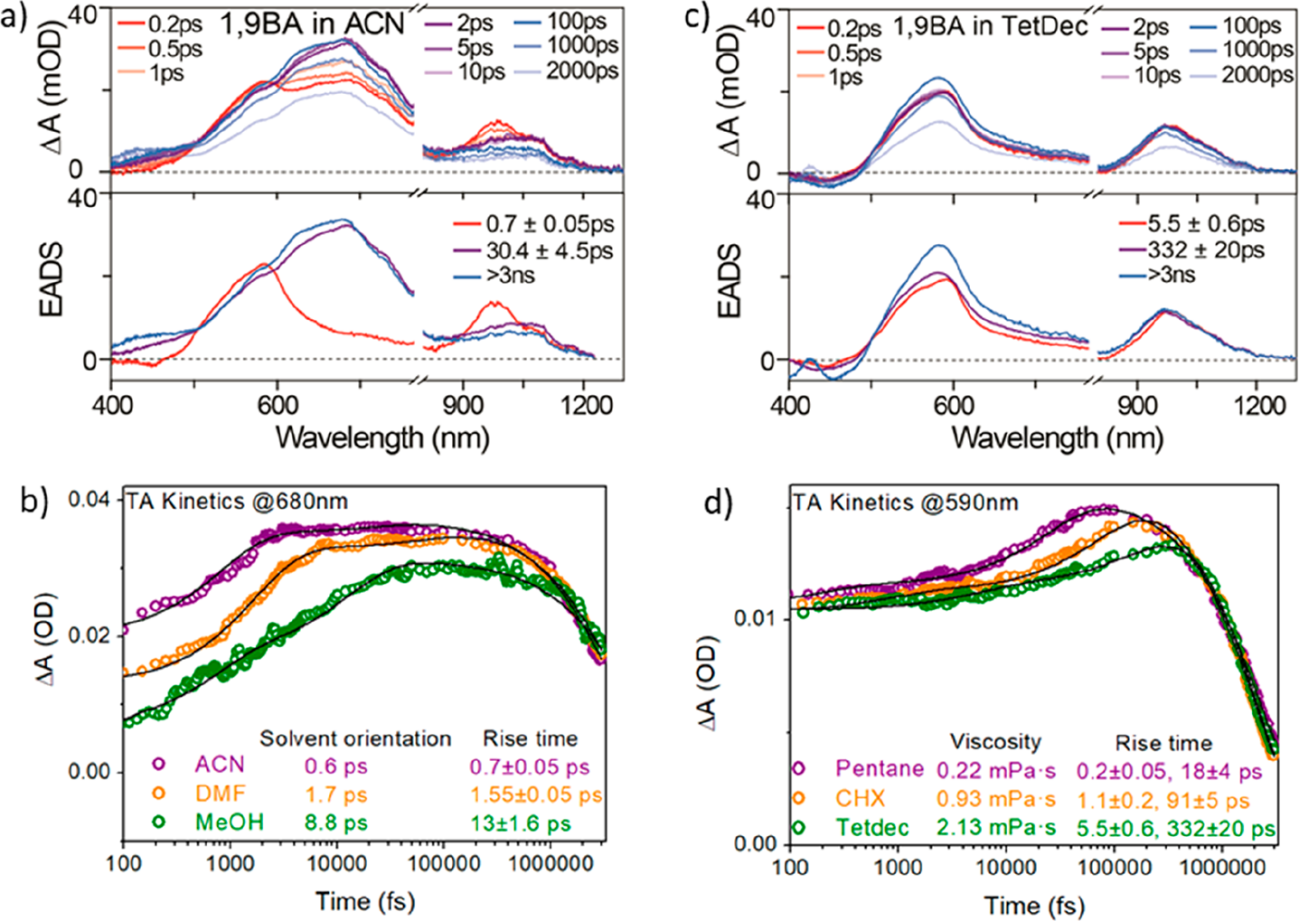
Transient absorption spectra and the evolution associated difference spectra (EADS) of 1,9′ BA in (a) acetonitrile and (c) tetradecane. A comparison of TA kinetics at (b) 680 nm in polar solvents and (d) 590 nm in nonpolar solvents is also shown.

Global analysis of the subnanosecond data required an initial state, an intermediate, and a final state (Figure 2a, bottom panel) to obtain a good fit; additional intermediates did not improve the fit quality or reveal any new spectral features. The dominant relaxation in acetonitrile is the 700 fs step from the LE FC state (590 nm) to the CS state (680 nm). The second relaxation, which occurs in tens of picoseconds, corresponds to a 7 nm blue-shift in the TA of the CS state. Incorporation of this tens of picoseconds relaxation step was essential for a good fit, and it was reproduced in other polar solvents (Figures S7 and S8). Such a slow relaxation in acetonitrile cannot be associated with solvation dynamics, which is much faster, and is thus assigned to intramolecular reorganization in 1,9′BA, leading to a stabilization of the CS excited state (and thus the observed blue-shift in S_1_ → S_*n*_ absorption). The tens of picoseconds time scale suggests that this reorganization involves large scale motion of the acene rings relative to one another.

No attempt was made to include the nanosecond decay to triplet state kinetics in the analysis (i.e., the appearance of the 450 nm TA) as data are sparse in that region. However, it is interesting to note that intersystem crossing from the CS singlet state leads to a triplet–triplet absorption characteristic of a LE state (Figure 2a), suggesting the triplet CS state is unstable with respect to the LE state even in polar solvents.

The same analysis was extended to the TA of 1,9′BA in polar solvents DMF and methanol. The data are shown in Figures S7 and S8. Data for methanol are of lower signal-to-noise because of low solubility. The CS state appears in 1.6 ps in DMF and 13 ps in methanol; both have longer CS times than the 0.7 ps for acetonitrile (Figure 2b). As previously reported by Lee et al. for 9,9′BA,^15^ these times correlate well with the orientational part of the solvation time correlation functions measured by Maroncelli: 0.6 ps for acetonitrile, 1.7 ps for DMF, and 8.8 ps for methanol (the latter being a weighted average of the two longer solvation times reflecting the complex orientational dynamics in the H-bonded liquid).^23^ This result confirms that CS is driven by solvent orientation relaxation in 1,9′BA, just as for 9,9′BA. Evidently, the CS kinetics are independent of the initial symmetry.

The ultrafast TA and EADS of 1,9′BA in nonpolar tetradecane are shown in Figure 2c (top and bottom panel, respectively). Once again, initial, intermediate, and final states are required to fit the subnanosecond dynamics, which are again followed by a nanosecond decay and formation of the LE triplet state (Figure 2c, top panel). The transient data and EADS (Figure 2c bottom panel) are, however, quite different than the polar solvent. In Figure 2c it is the faster (5.5 ps) relaxation which involves the subtle change in spectra; in this case a small blue-shift and loss of structure result from evolution of the FC to a new LE state. This is followed by an unexpected increase in the amplitude of the transient absorption at 585 nm on the hundreds of picoseconds time scale. This slow relaxation must indicate evolution of the rapidly formed LE intermediate to a new excited state with enhanced S_1_ → S_*n*_ oscillator strength. The behavior in Figure 2c was highly reproducible across four nonpolar solvents with different viscosities, as shown in the Figures S4–S6. These nonpolar dynamics are assigned to an excited state structural relaxation to the distorted slightly polar structure suggested by TD-DFT calculations (Figure 1b), specifically a small ring distortion and 27° twisting of the anthracene rings relative to one another. A similar increase in transient absorption amplitude was observed for 2,9′BA in nonpolar solvent but was considered to be associated with formation of a CS state.^11^ Here and through FSRS (below) we show that the dynamics and spectroscopy of the intermediates in polar and nonpolar solvents are very different. The NIR TA also has a signature of this excited state twisting reaction, in this case a tens of nanometers blue-shift (Figure 2c).

TA for 1,9′BA was measured and analyzed in a series of nonpolar solvents of varying viscosity (Figures 2d and S4–S6). Plots of the rate constant for the two steps observed in the global fitting ( are a linear function of *a*(*T*/η), where *a* is a proportionality constant, η is the viscosity of solvent, and *T* is the temperature (Table S4 and Figure S10). The linear (*T*/η) dependence suggests that the picosecond excited state dynamics of 1,9′BA in nonpolar solvent are under diffusion control. These data may be analyzed further using the model of Oster and Nishijima for excited state relaxation occurring via diffusive rotational motion on a flat excited state potential energy surface.^24,25^ If a rotating sphere of volume *V* undergoes an angular displacement of ΔΘ, then ΔΘ^2^ = *k*_B_*T*/(3*aV*), where *k*_B_ is Boltzmann’s constant. Using the *a* value calculated for the slower relaxation time and assuming ΔΘ to be 27° (from the DFT/TDDFT calculation), we recover the hydrodynamic volume of the rotating particle as 54 Å^3^. This should correspond to the hydrodynamic volume of the rotating body (here the anthracene unit). The value recovered is within a factor of 3 of that obtained for orientational relaxation of anthracene alone.^26^ Given the inherent approximation on shape, PES, and freedom of movement, this result is consistent with diffusion control of the slower relaxation component in nonpolar solvents. Clearly the faster relaxation time displaces a much smaller volume, and we speculate that this may be associated with the distortion coordinate (although other factors may contribute on such a fast time scale).

To obtain structural insight into the solvent-dependent excited state dynamics of 1,9′BA, tunable femtosecond stimulated Raman spectroscopy (FSRS) was applied to record vibrationally resolved spectra of its distinct excited states. FSRS measures the stimulated Raman spectrum of resonant transient electronic states through a combination of narrowband Raman pump and broadband femtosecond probe pulses as a function of time after excitation by an “actinic” pump pulse.^27,28^ The method, apparatus, and data analysis procedures have been described elsewhere and are detailed in the Supporting Information and Figure S11.^29,30^ The data for 1,9′BA in nonpolar tetradecane (selected for its relatively weak nonresonant solvent contribution in FSRS) are shown in Figure 3a. The Raman pump wavelength was set at 560 nm, resonant with the LE state transient absorption, while avoiding overlap with the stimulated emission, which can complicate line shapes in FSRS.^31^ The nonpolar solvent spectrum is dominated by a single slightly asymmetric signal at 1030 cm^–1^. This can be assigned as arising from the FC LE state, as it appears at *t* = 0. By shifting the Raman pump wavelength to 650 nm, the spectrum is unchanged but greatly reduced in amplitude (Figure S12), which is ascribed to the weaker resonance enhancement (Figure 2c). The amplitude of the 1030 cm^–1^ mode decays monotonically, with triexponential kinetics, a dominant 500 fs decay, and then longer components of ca. 8 and 80 ps (Figure 3b). Note that a decay in amplitude is occurring even though the resonant TA being probed is increasing in amplitude with time (Figure 2c). Thus, we assign the 560 nm Raman pump signal to the FC LE state, which decays in 500 fs to form a partially distorted intermediate state. This distorted state must have a significantly lower resonant Raman cross section than the FC LE state. It then undergoes further relaxation under diffusion control (see above) to form the final relaxed geometry on the tens to hundreds of picoseconds time scale (i.e., we identify the ca. 8 and 80 ps components in FSRS with the 5 and 300 ps time constants determined in TA (Figure 2d); TA time constants are more accurate because a higher signal-to-noise is obtained with a greater density of data over a wider time range). The persistence of the 1030 cm^–1^ signal on these longer times suggests that an equilibrium is established between relaxed and FC geometries, which in turn suggests they are separated by only a small potential energy barrier on the order of *kT*. To compare with the ground state modes, the ground state spontaneous Raman (GSR) spectrum of 1,9′BA was recorded using a 532 nm excitation laser. The strongest mode in the ground state arises at around 1400 cm^–1^, corresponding to the C=C stretching frequency (Figure S13). However, this mode is absent in the excited state Raman spectrum, which has no obvious corresponding ground state mode.

**Figure 3:**
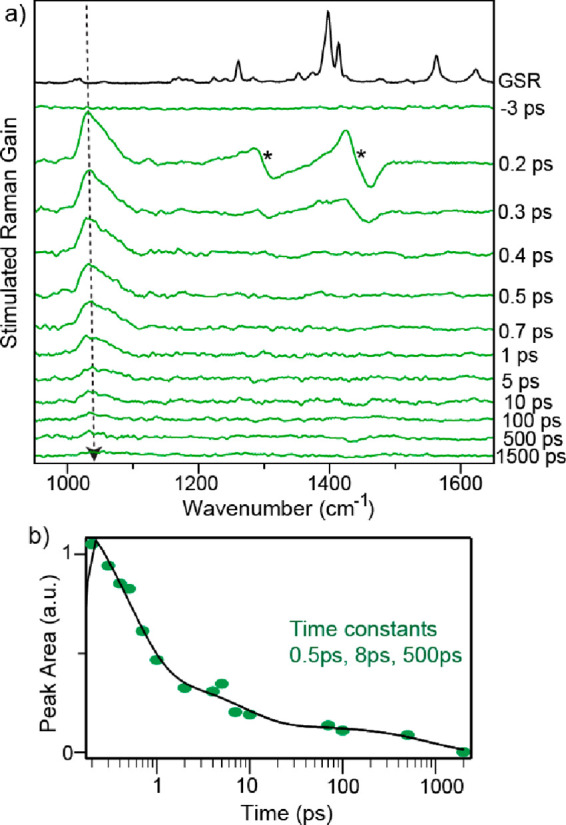
(a) Transient stimulated Raman spectra at different time delays and (b) the peak area kinetics of 1,9′BA in tetradecane with a Raman pump at 560 nm. GSR represents the ground state spontaneous Raman spectrum of 1,9′BA, and the asterisk (∗) symbol shows the solvent artifact.

In order to assign the dominant mode at 1030 cm^–1^, TD-DFT calculations were performed (using conductor-like polarizable continuum model (CPCM) for cyclohexane) on both the FC geometry (i.e., equivalent to the ground state geometry) and a geometry where the torsional angle was fixed at 90° but the structure was otherwise allowed to relax. These unoptimized geometries are chosen as the 1030 cm^–1^ mode is detected in the FC state before the relaxed LE state forms in a diffusion-controlled process (as shown in TA, above). Both calculations yielded a negative frequency associated with torsional motion of the anthracene rings relative to one another, i.e., the reaction coordinate. The 1000–1100 cm^–1^ spectral region in the calculated S_1_ Raman spectrum reveals five CH in-plane bending modes, two of which are coupled to inter-ring C–C stretching (see SI Figure S15). Modes which exhibit strong resonance Raman enhancements are often those which show a significant displacement between resonant states. The TD-DFT calculation suggest the inter-ring CC bond shortens between S_0_ and the relaxed S_1_ state making these two modes’ plausible assignments. Further, the calculations show that one mode (at 1038 cm^–1^) is relatively strong in the unoptimized FC state but weak in the relaxed but torsionally constrained calculation (Figure S15). This would therefore be consistent with the observation that the 1030 cm^–1^ mode decays on the subpicosecond time scale rather than the picosecond torsional relaxation (Figure 3b).

However, any such assignment must be tentative because the large enhancement at 1030 cm^–1^ must arise through resonance between the FC state and a higher lying S_*n*_ state, not calculated here. Thus, while the measured frequency indeed reflects an S_1_ FC state mode, the intensity reflects the upper state properties.^32,33^ The calculation of such enhancements requires knowledge of the nature of the S_*n*_ state and especially the mode displacements between FC and S_*n*_.^33,34^ These are difficult to predict. For example, if the S_1_ → S_*n*_ transition involved inter-ring charge transfer excitation character, the CC stretch might modulate that transition and so be strongly enhanced. In the absence of such detailed calculations, ideally supported by FSRS measurements on other transitions (e.g., in resonance with the NIR TA, Figure 2c), a more robust assignment of this mode is not possible; all we can firmly conclude at present is that the enhanced 1030 cm^–1^ mode is a marker band for the FC LE state.

In Figures 4a and 4c the FSRS spectra for 1,9′BA in acetonitrile are shown for resonant wavelengths of 560 and 650 nm (i.e., the LE and CS state’s TAs, respectively). The time-dependent amplitudes are included in Figures 4b and 4d. The characteristic 1030 cm^–1^ FC LE state mode is recovered with the 560 nm Raman pump and decays in 500 fs. This could reflect either FC state decay (as seen in tetradecane) or solvation driven CS as seen in TA, or a combination of both. However, it is significant that in acetonitrile the 1030 cm^–1^ FSRS amplitude decays to the baseline rather than to a long-lived intermediate as in tetradecane (compare Figures 3b and 4b) such that the twisted intermediate is evidently not populated. This suggests that the LE FC state decays directly to the CS state through solvent orientational relaxation rather than via the twisted structure formed through diffusion.

**Figure 4:**
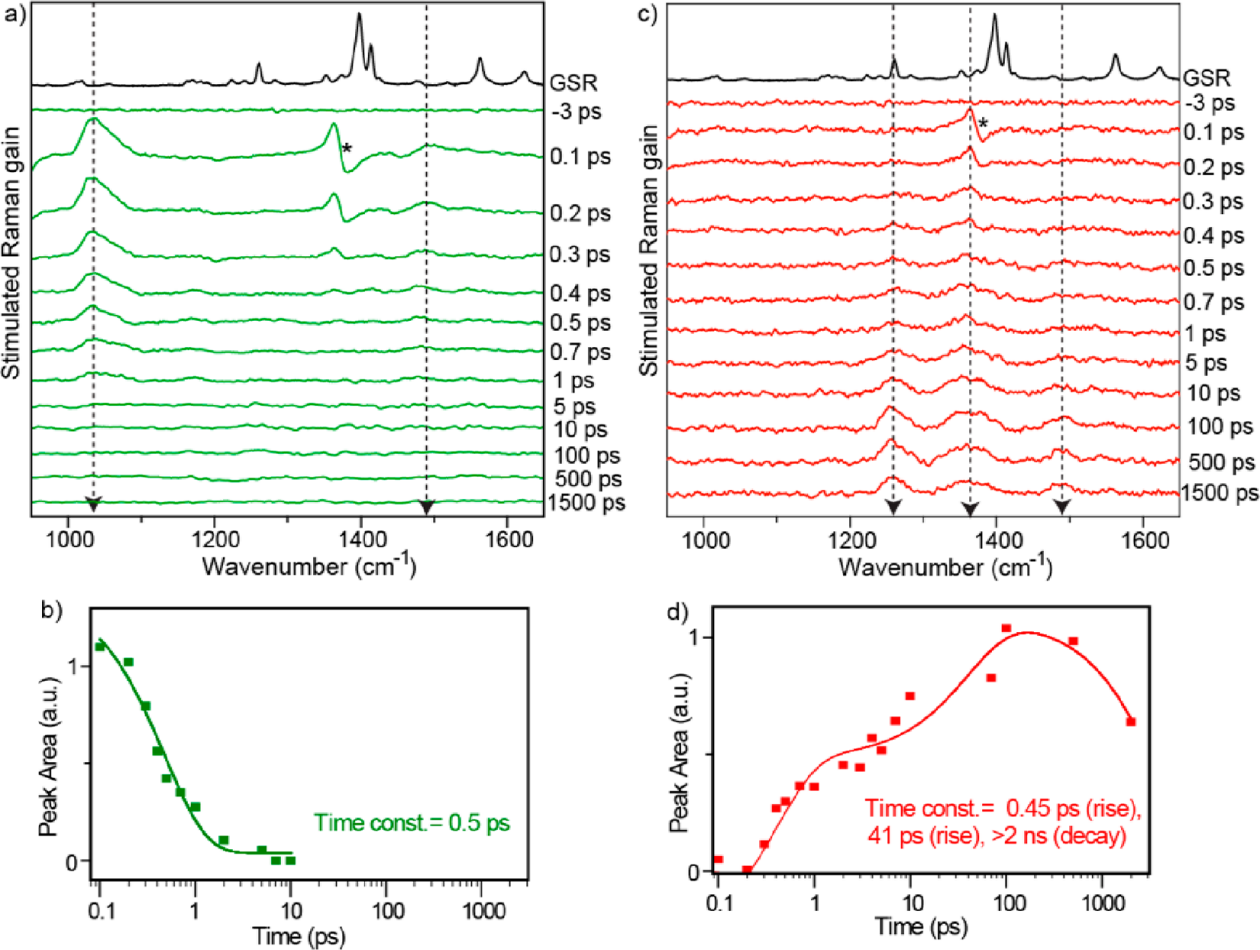
Transient stimulated Raman spectra at different time delays and the corresponding peak area dynamics of 1,9′BA in acetonitrile with a Raman pump at (a, b) 560 nm and (c, d) 650 nm. GSR represents ground state spontaneous Raman spectrum of 1,9′BA, and the asterisk (∗) symbol the shows solvent artifact.

The FSRS spectrum measured at 650 nm (resonant with the CS state) reveals a quite different spectrum with four distinct modes, which are absent at *t* = 0. This spectrum forms with an initial 450 fs rise time (i.e., matching the LE FC state decay and consistent with a solvation-controlled CS) and then shows a further growth on a tens of picoseconds time scale (Figure 4c,d), reflecting the slow structural reorganization resolved in TA of the CS state (Figure 2a). Significantly, these four modes at 1255, 1362, 1490, and 1550 cm^–1^ are very similar to calculated ground state resonance Raman frequencies for the anthracene radical anion and cation (see Figure S14). The DFT calculation using the CPCM model for the acetonitrile solvent shows modes at 1240, 1360, 1495, and 1530 cm^–1^ which arise from different ring breathing modes of the anthracene radical cation or anion rings (see Figure S16). These may be compared with earlier steady state Raman data for dimethylanthracene radicals (here averaged for the anion and cation) in their electronic ground state at 1194, 1280, 1380, and 1560 cm^–1^.^21^ These results thus support the CS nature of the final state of 1,9′BA in polar solvents and that it is formed on the time scale of solvation.

In summary, these data reveal complex solvent-dependent excited state dynamics in 1,9′BA, with distinct behavior in nonpolar and polar solvents; these are represented in Figure 5. In nonpolar solvents the FC state is populated upon excitation of the 90° twisted ground state. The FC state is characterized by a strong resonance Raman spectrum dominated by a single mode tentatively assigned to CH bend plus inter-ring CC stretch. This FC state relaxes in 500 fs to form an intermediate state with a lower Raman cross section. This metastable state relaxes in a diffusion-controlled torsional relaxation to a state which, according to both TD-DFT calculation and analysis of the viscosity dependence, has a structure with anthracene rings twisted to a torsion angle of ca. 63°. Diffusion control suggests that the slower step displaces a significant volume of solvent, and that motion is along a flat potential energy surface. As a result, the emissive state in nonpolar solvents will be energetically close to the FC state, possibly within *kT*, such that this state is in thermal equilibrium with the FC state. A similar excited state structure change was suggested for 9,9′BA although its dynamics were not resolved.^15,16^

**Figure 5:**
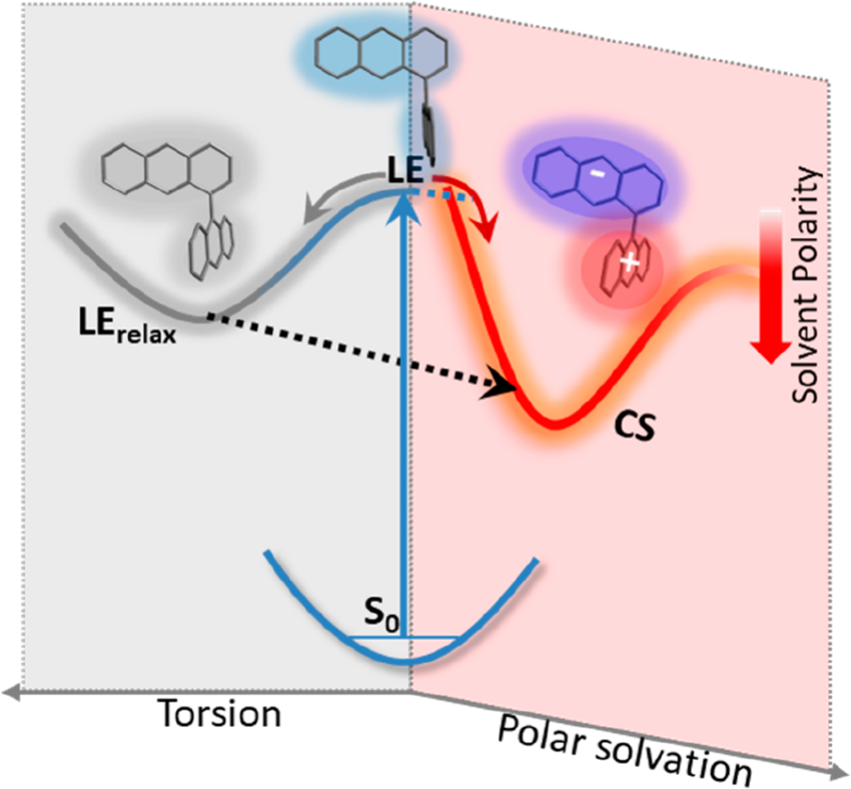
Potential energy diagram showing evolution from the local excited state (LE) to either the relaxed LE or charge separated state (CS) depending on the nature of solvent. In nonpolar solvent the FC LE state relaxes in 500 fs to a distorted structure which goes on to a slightly polar twisted LE_relax_ state in a diffusive process. The relaxed state is in equilibrium with the initial FC state. In polar solvent the FC state undergoes solvation driven relaxation directly to the CS state without going through the distorted intermediate. However, the polar LE_relax_ state could act as a driving force for CS in less polar or slowly relaxing solvents.

At its energy minima this twisted excited state structure is calculated to have a nonzero dipole moment. Thus, the intramolecular reorganization in nonpolar solvents is a potential source of a symmetry breaking electric field in BAs, where the initially small molecular dipole moment in the twisted state can initiate polar solvation, which can then in moderately polar or slowly relaxing polar solvents drive the full CS; this is represented by the black dashed line in Figure 5. The small dipole moment may be fluctuating on a picosecond time scale due to the low stabilization energy relative to the 90° FC state geometry.

In strongly polar solvents both dynamics and spectroscopy show that CS state formation is dominated by orientational solvation dynamics, as previously shown for 9,9′BA. This pure solvation control shows that in fast relaxing solvents direct CS occurs from the FC state on the time scale of solvation without going through the twisted intermediate; the instantaneous asymmetry in electric field is provided by the fluctuating solvent orientational polarization. The time-dependent Raman spectra of this nanosecond lifetime state are consistent with formation of anthracene radicals, confirming the CS reaction. Finally, both distorted LE states and CS states decay to a LE like triplet through intersystem crossing.

## Experimental Details

### Synthesis of 1,9′BA

In a glovebox, a solution of anthracene-9-boronic acid (0.20 g, 0.79 mmol), 1-iodoanthracene (0.20 g, 0.66 mmol), Pd(PPh_3_)_4_ (23.0 mg, 19.8 μmol), and CsF (0.36 g, 2.37 mmol) in dry DME (8 mL) was prepared in a sealed tube. The mixture was stirred and heated at 85 °C for 72 h. The reaction mixture was cooled to rt and filtered through a silica plug using DCM, and the solvents were evaporated. The residue was purified by silica gel chromatography using petroleum ether/DCM (30:1 to 5:1). Recrystallization from MeOH/DCM afforded 1,9BA (196 mg, 84%) as a yellow-biege solid; mp 253–255 °C. ^1^H NMR (400 MHz, methylene chloride-*d*_2_) δ: 8.66 (s, 1H), 8.62 (s, 1H), 8.25 (d, *J* = 8.7 Hz, 1H), 8.15 (d, *J* = 8.7 Hz, 2H), 8.03 (d, *J* = 8.7 Hz, 1H), 7.70 (dd, *J* = 8.7, 6.7 Hz, 1H), 7.61 (s, 1H), 7.51 (dd, *J* = 6.7, 1.2 Hz, 1H), 7.50–7.39 (m, 6H), 7.27 (ddd, *J* = 8.7, 6.7, 1.2 Hz, 1H), 7.22 (ddd, *J* = 8.7, 6.7, 1.2 Hz, 2H). ^13^C NMR (101 MHz, chloroform-*d*) δ: 136.71, 135.09, 131.97, 131.80, 131.67, 131.53, 131.14, 128.63, 128.55, 128.45, 128.42, 127.86, 127.01, 126.64, 125.60, 125.55, 125.25, 125.20, 125.10. ^1^H and ^13^C spectra are presented in Figures S17 and S18.

**Scheme 1:**
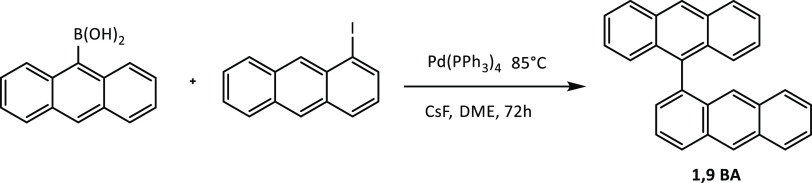
Synthesis of 1,9′BA

### Ultrafast Spectroscopy

The TA and FSRS apparatus have been described in detail elsewhere,^29,35^ and specific details are presented in the Supporting Information.

### DFT and TD-DFT

The ground state geometry and frequency were optimized using the DFT method with the B3LYP functional, 6-311G(2d,2p) basis set, and solvent model with conductor-like polarizable continuum mode (CPCM). The excited state geometry and frequency optimization were performed using the TDDFT method with restricted cam-B3LYP functional, 6-31G(d,p) basis set, and CPCM model. Details are presented in the Supporting Information.

## Supporting Information Available

Details of the experimental methods, additional figures and tables specifically: experimental details on absorption, fluorescence, transient absorption and femtosecond stimulated Raman; details of computation of excited and ground state structures by DFT and TDDFT; data analysis and global fitting; temperature-dependent emission spectra; tables of measured spectral transition wavelengths as a function of solvent; table and figure of fluorescence lifetimes; figures and analysis of solvent-dependent transient absorption; table of data for rotational diffusion analysis and associated graphs; details of treatment of femtosecond stimulated Raman data; details of DFT and TDDFT calculations of Raman spectra; atomic coordinated from DFT and TDDFT calculations; NMR spectra of the synthesized 1,9BA (PDF)
Transparent Peer Review report available (PDF)
