# Supplementary material for: Solvent Tuning Excited State Structural Dynamics in a Novel Bianthryl
Source: J Phys Chem Lett. 2023 Jan 3;14(1):253–9. doi: 10.1021/acs.jpclett.2c03469 (PMC9841557; doi:10.1021/acs.jpclett.2c03469)
Supplement: Supplementary file 1 — jz2c03469_si_001.pdf [file jz2c03469_si_001.pdf]

SUPPLEMENTARY INFORMATION

Solvent Tuning Excited State Structural Dynamics in a Novel Bianthryl

Palas Roy,[1] Faisal Al-Kahtani, Andrew N. Cammidge and Stephen R. Meech*

School of Chemistry, University of East Anglia, Norwich NR4 7TJ, UK

[1]Present address: School of Basic Sciences, Indian Institute of Technology Bhubaneswar, Odisha 752050, India

S1 Experimental and Computational Detail

Figure S1. Temperature dependent emission spectra of 1,9'BA

Figure S2. Lippert-Mataga plot

Figure S3. Time-resolved emission spectra of 1,9'BA

Figure S4-S8. Transient absorption spectra and the evolution associated difference spectra

Figure S9. TA data of 1,9'BA corresponding to long-lived component

Figure S10. Rotational diffusion analysis

Figure S11. Raw excited state Raman data

Figure S12. FSRS spectra of 1,9'BA in tetradecane at different time delays

Figure S13. Spontaneous Raman

Figure S14-15. TDDFT Calculated S_1 Raman spectra

Figure S16. DFT Calculated Raman spectra of radicals

Figure S17. ^{1}H NMR Spectrum of compound 1,9'BA

Figure S18. ^{13}C NMR Spectrum of compound 1,9' BA

Figure S19. Emission spectra for quantum yield measurement

Figure S20. Oxidation of anthracene and 1,9' BA

Table S1. Absorption and emission maxima of 1,9'BA

Table S2. Table values for Lippert-Mataga plot

Table S3. Time-resolved emission

Table S4. Table for rotational diffusion analysis

Nuclear coordinates from calculation

References

Experimental Details

Steady-state absorption: Steady-state absorption spectra were recorded using a PerkinElmer Lambda XLS spectrophotometer with a 1 cm path length quartz cuvette.

Time-resolved and steady-state fluorescence: Steady-state Fluorescence spectra of 1,9'BA in different solvents were recorded using Edinburgh Instruments FS5 Spectrofluorometer equipped with continuous (150 W) xenon lamp where the excitation wavelength used was 380 nm. Time-resolved fluorescence dynamics were measured under the excitation of a 365 nm picosecond laser pulse using the spectrometer based on a time correlated single photon counting (TCSPC) technique. The lifetimes were determined via the equation: y (t) =c $\sum_{i=n}$ a_i exp(-t/τ_i) X Gaussian IRF, where c is scaling factor, a_i is amplitude of the exponential, τ_i is the time constant and Gaussian IRF is the instrument response function. 1cm path length cuvette was used for all the measurements while keeping sample absorbance below 0.2 OD.

Emission quantum yield measurements: The emission quantum yield (Q) of the 1,9'BA in CHX was determined using anthracene monomer as reference standard with a quantum yield of 0.36.(REF: Berlman, 1971) Concentrations of the samples were kept around 0.065 OD (at 350 nm) in a 1 cm cell. The emission spectra for sample and reference were collected at 350 nm excitation (SI Figure S19) keeping all the parameters (data accumulation time, slit width) the same. Yields were calculated according to:

$$Q_f = Q_r \cdot \left(\frac{I_f}{I_r}\right) \cdot \left(\frac{A_r}{A_f}\right) \cdot \left(\frac{n_f^2}{n_r^2}\right)$$

Where Q_f = fluorescence quantum yield of 1,9'BA; Q_r= quantum yield of Anthracene reference; A= absorbance (at 350 nm); I = integrated emission area; n = refractive index. The recovered quantum yield was 0.54.

Femtosecond transient absorption measurements (TA): Details about transient absorption set up used here has been described elsewhere.[1] In brief, TA is a two pulse technique – a femtosecond pump pulse excites the molecules electronically and then a time delayed broadband white light continuum pulse probes the resultant changes in the electronic spectra. A regenerative Ti:sapphire amplifier (Spectra Physics Spitfire ACE) is used to amplify the fundamental beam from the Spectra Physics Mai Tai laser oscillator and to generate output pulses centered at 800 nm with duration of

120 fs, repetition rate of 1 kHz and energy of 5 mJ per pulse. This amplified laser output is then used to drive two commercial optical parametric amplifiers (OPA, Light Conversion TOPAS Prime). One OPA is used to generate the 390 nm pump pulse with duration of 80 fs in order to excite the bianthryl samples. The pump pulse was passed through a mechanical chopper (500 Hz) and a computer-controlled delay stage. A combination of half wave plate and a polarizer was used to set polarization at magic angle. The second OPA is tuned to generate the 1250 nm pulse which was focused on to a 3 mm thick sapphire window to generate broadband white light continuum (500-1400 nm). To access the WLC probe in the 400-800 nm spectral windows, we directly focused the fundamental 800 nm beam onto the sapphire plate. All the TA Spectra were measured in two separate overlapping WLC probe windows and then stitched together.

A 50/50 beam splitter was used to split the WLC beam before the sample stage. One part (Reference WLC) was used in the reference detection channel to correct for intensity fluctuations in the probe spectrum. The second part (Probe WLC) was spatially and temporally overlapped with the pump pulse in a 1 mm thick sample cell which had 0.5 mm thick fused silica windows. The focal spot sizes of the pump and probe pulses were 250 µm and 50 µm respectively. Pump beam energy was attenuated to 0.2 mW at the sample cell. After passing through the sample stage the probe beam was aligned collinearly with the reference beam and on top of each other. Both the beams were dispersed using a home-built prism based spectrograph and then focused and detected by two separate synchronised 16 bit A/D CCD detectors from Entwicklungsbüro Stresing (1024 pixels). A chopper at 500Hz in the path of pump beam allowed the detection of pump-on/pump-off probe and reference spectra. The referenced difference spectrum for each pulse pair was calculated using:

$$\Delta A = -log(\frac{Probe\ (Pump\ on)\ \mathrm{X}\ Refernce\ (Pump\ off)}{Reference\ (Pump\ on)\ \mathrm{X}\ Probe\ (Pump\ off)})$$

Mercury-Argon lamp (HG-1 Ocean Optics) was used to calibrate the detector. The solvent Kerr response with 390 nm excitation pump and probe beams provide the instrument response function (IRF) to be about 100 fs. The data presented were average over 5 cycles and each time trace was accumulated for 0.1 sec. All pump-probe measurements were done in 1 mm cell under flowing condition with the liquid driven by a peristaltic pump (World Precision Instruments) in order to avoid any photodamage.

Global Analysis: The TA dataset were analyzed using Global analysis (Glotaran 1.5.1 software package),[2] which reports both the Evolution Associated Difference Spectra (EADS) and lifetimes associated with each species. Here we used three states connected in a sequential scheme: A → B

→ C. In the Supplementary Figures S4-S8, we show evolution associated decay spectra for 1,9'BA in both polar and nonpolar solvents. It shows three time components corresponding to A, B and C. The good quality of fit has been verified by looking at the wavelength dependent kinetic fittings (see main Figure 2b, 2d).

Femtosecond stimulated Raman measurements (FSRS): The same setup used for TA measurement was modified for the FSRS measurements and has been described elsewhere.[3] Briefly, FSRS is a three-pulse technique: femtosecond actinic pump, Raman pump and white–light probe pulse. The 'actinic' pump excites the stable ground state molecule in electronically higher excited state. Then the sample is probed by a broadband femtosecond probe pulse in the presence of a narrowband (10 cm^{-1}) picosecond Raman pulse, which together stimulate the coherent Raman scattering process from the sample. The white-light continuum probe (generated using 1250 nm OPA output) and actinic pump pulses were generated as described in the TA section. On the other hand, the Raman pump pulse was generated by sending part of the amplified fundamental beam (from Spectra Physics Spitfire ACE amplifier) through a commercial second harmonic bandwidth compressor (SHBC from Light Conversion) and then a picosecond configured OPA (TOPAS-PS from Light Conversion). All the three beams- Actinic pump, Raman pump and white–light probe pulse are focused (spot sizes 250, 150 and 50 μm respectively) and overlapped spatially and temporally inside the 1mm sample cell. No reference Probe was used for the FSRS measurements. Stimulated Raman from cyclohexane was used to optimize the overlap between Probe and Raman pump pulses. Delay between actinic pump and Raman probe is controlled by a computer-controlled delay stage. The transmitted probe beam was dispersed in a high spectral resolution (<10 cm^{-1}) grating spectrometer (SPEX 500M) and the probe was detected using a single CCD (1024 pixel). The detector was calibrated using Raman peaks of cyclohexane. Spectral resolution as determined from the line width of the cyclohexane 802 cm^{-1} peak is about 10 cm^{-1}. Each spectrum was accumulated for 6 s and a total of 10 scans were recorded using LabView controlled software. The actinic pump power used was 1 mW at 390 nm for 1,9'BA in all the solvents. The Raman pulse was tuned at 560 and 650 nm (4 mW) to be in resonance with the excited state absorption of 1,9'BA. Raman pump at 650 nm is in resonance with the charge separated state while that at 560 nm is in resonance with the local excited state. All the FSRS measurements were done in both polar acetonitrile and nonpolar tetradecane under similar experimental conditions (overlap, power, concentration) so that we can compare the effect of solvent polarity. The sample absorbance (at 390nm) was kept at 0.5 OD during all measurements. The sample was circulated continuously through a 1 mm flow sample cell with the liquid driven by a peristaltic pump to replenish fresh molecule for each pump pulse. Time resolution of the experiment

was determined from the solvent response dictated by the actinic pump and probe pulse convolution which is about 100 fs.

Spectra were recorded at 1 kHz. The Raman pump and actinic pulses were passed through two synchronized mechanical choppers operating at 250 Hz and 500 Hz respectively. This result in four different signals: i) Excited state FSRS (Probe + Raman + Actinic); ii) Transient absorption (Probe + Actinic); iii) Ground state FSRS (Probe + Raman) and iv) Probe reference (Probe only). The unprocessed FSRS signal is obtained when Actinic pump, Raman pump and probe pulses are present on the sample as we detect log ($I_{Raman+Actinic+Probe}/I_{Probe}$). Along with excited state Raman, this unprocessed FSRS signal also contains information on ground state Raman (log ($I_{Raman+Probe}/I_{Probe}$)), transient absorption (TA) (log ($I_{Actinic+Probe}/I_{Probe}$)) and nonlinear background. Therefore the unprocessed FSRS data has been subtracted from its ground state Raman and TA contributions. In our experiment, the ground state Raman signal has large solvent contributions which is also subtracted. The resultant difference spectra (say, raw excited state Raman signal (ESR_{RAW})) are plotted in the Supplementary Figure S11. These are baseline corrected to obtain the raw excited state Raman signal as shown in main Figure 3 and 4. This same procedure is followed for different pump-probe time delays.

Computational details: All DFT and TDDFT calculations were carried out using Gaussian16.[4] The ground state geometry optimization and non-resonant Raman spectra calculations were optimized using DFT method with B3LYP functional, 6-311G(2d,2p) basis set and solvent model with conductor-like continuum mode (CPCM). The excited state geometry and frequency optimization were carried out using TDDFT method with restricted cam-B3LYP functional, 6-31G(d,p) basis set and CPCM model. All frequencies have been scaled by a factor of 0.985 (to account for a systematic error in the DFT predictions).

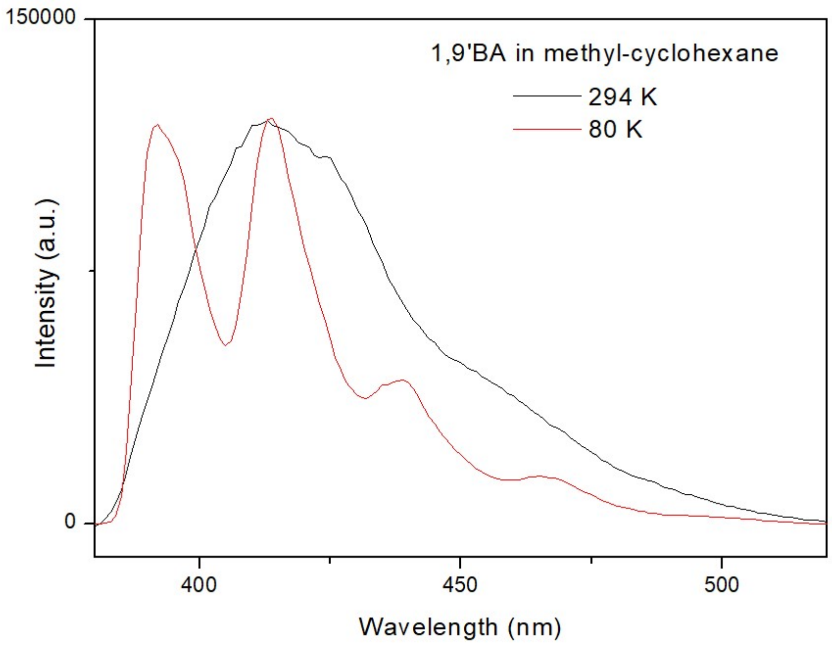

Figure S1. Temperature dependent emission spectra of 1,9’BA showing structural mirror image emission at <80K.

	Toluene	Cyclohexane	TetDec	Methyl-THF	MeOH	DMF	ACN
Abs. max. of 1,9BA	389nm	386nm	387nm	387nm	385nm	389nm	386nm
Em. max. of 1,9BA	418nm	410nm	410nm	429nm	452nm	467nm	471nm

Table S1. Absorption and emission maxima of 1,9'BA in different solvents

Solvents	Dielectric constant (ε)	Refractive index (n)	Δf	Abs. maxima /nm	Emi. maxima /nm	$\Delta\tilde{\nu}$ /cm^{-1}
Cyclohexane	2.02	1.4262	-0.00165	386	411	1576
Toluene	2.38	1.4969	0.01324	389	418	1783
Methyl-THF	7.58	1.4072	0.20957	387	429	2530
Acetonitrile	37.5	1.3441	0.30542	386	471	4675
DMF	36.7	1.4305	0.27438	389	467	4294
Methanol	32.7	1.3284	0.30859	385	452	3850

Table S2. Table values for Lippert-Mataga plot of emission peak shift

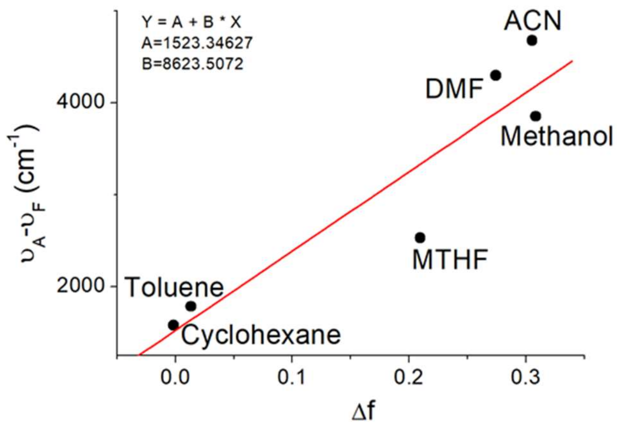

Figure S2. Lippert-Mataga plot of emission peak shift as a function of solvent polarity parameter

	Cyclohexane	TetDec	Toluene	Methyl-THF	MeOH	DMF	ACN
Emission Kinetics at	410 nm	410 nm	450 nm	450 nm	470 nm	470 nm	470 nm
Lifetime values	3.612 ± 0.007 ns	3.72 ± 0.006 ns	4.221 ± 0.007 ns	5.366 ± 0.01 ns	8.09 ± 0.017 ns	12.01 ± 0.023 ns	8.69 ± 0.019 ns

Table S3. Time-resolved emission wavelength and the lifetime values of 1,9'BA

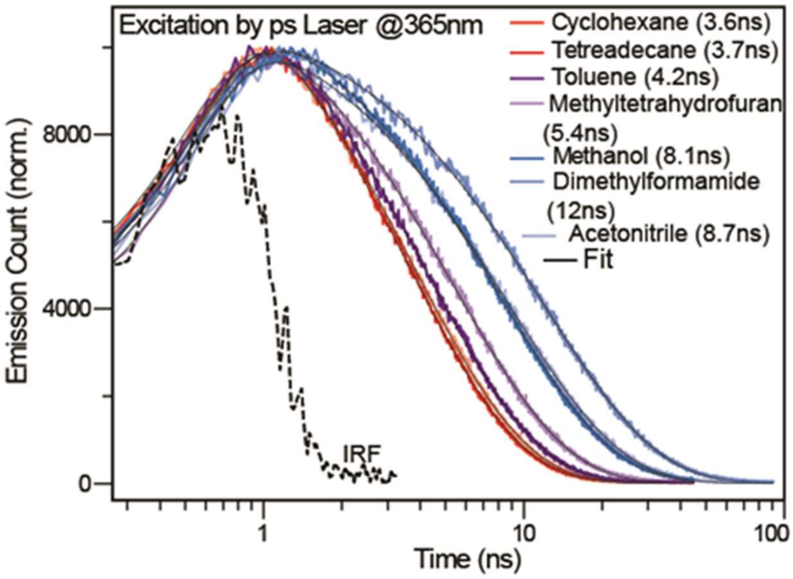

Figure S3. Time-resolved emission spectra of 1,9'BA using excitation by laser pulse at 365 nm

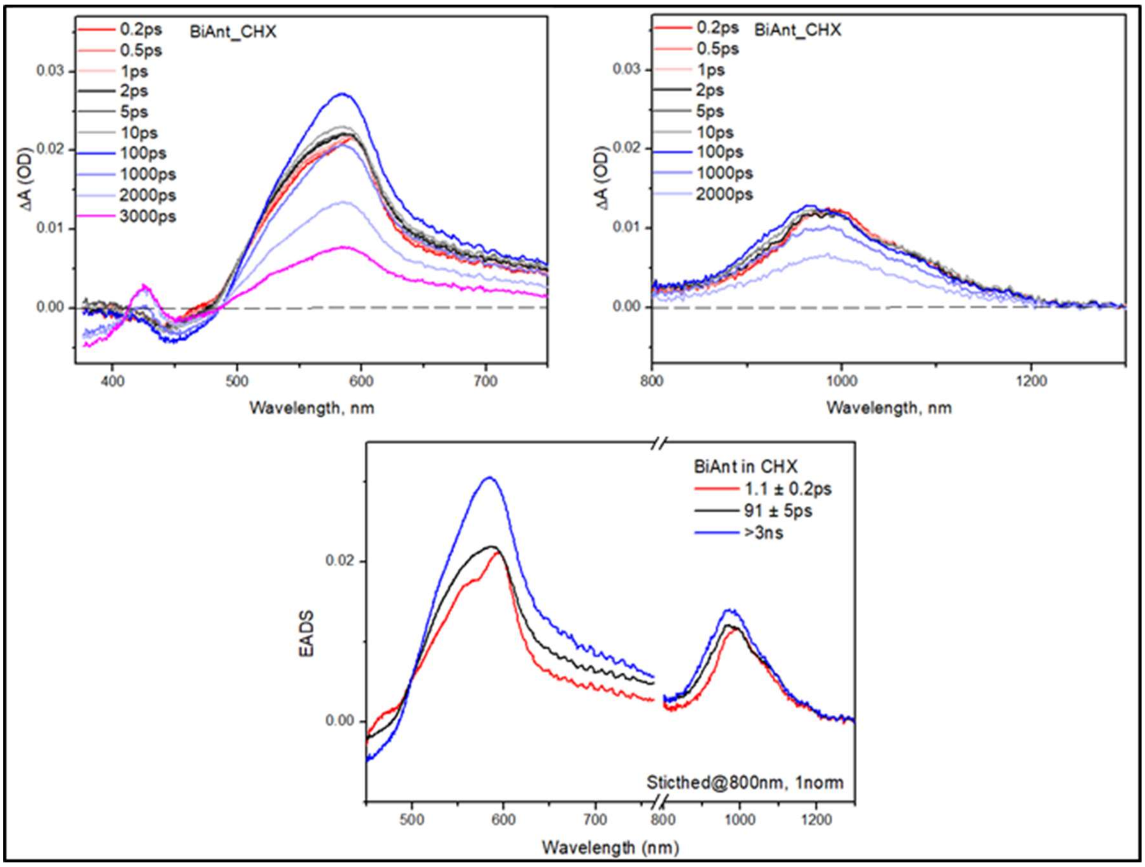

Figure S4. Transient absorption spectra and the evolution associated difference spectra (EADS) of 1,9' BA in cyclohexane

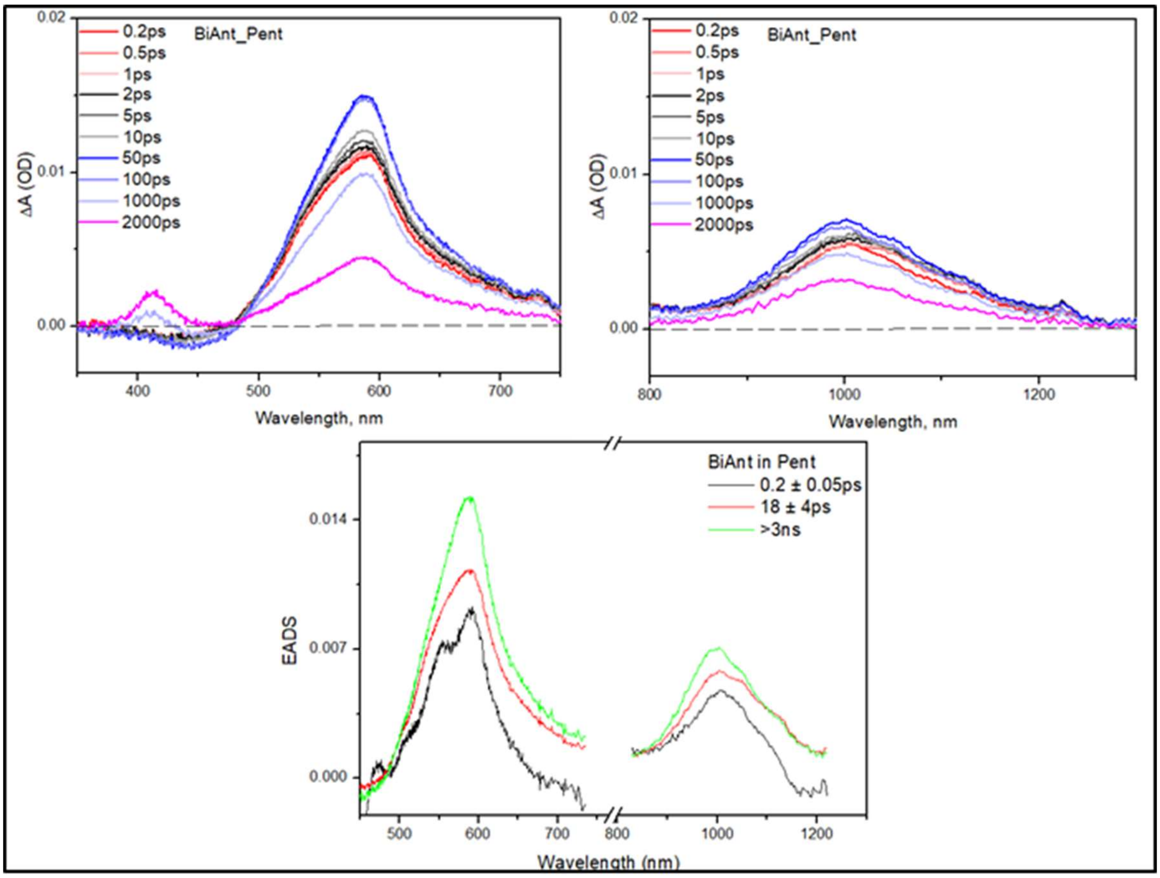

Figure S5. Transient absorption spectra and the evolution associated difference spectra (EADS) of 1,9' BA in pentane

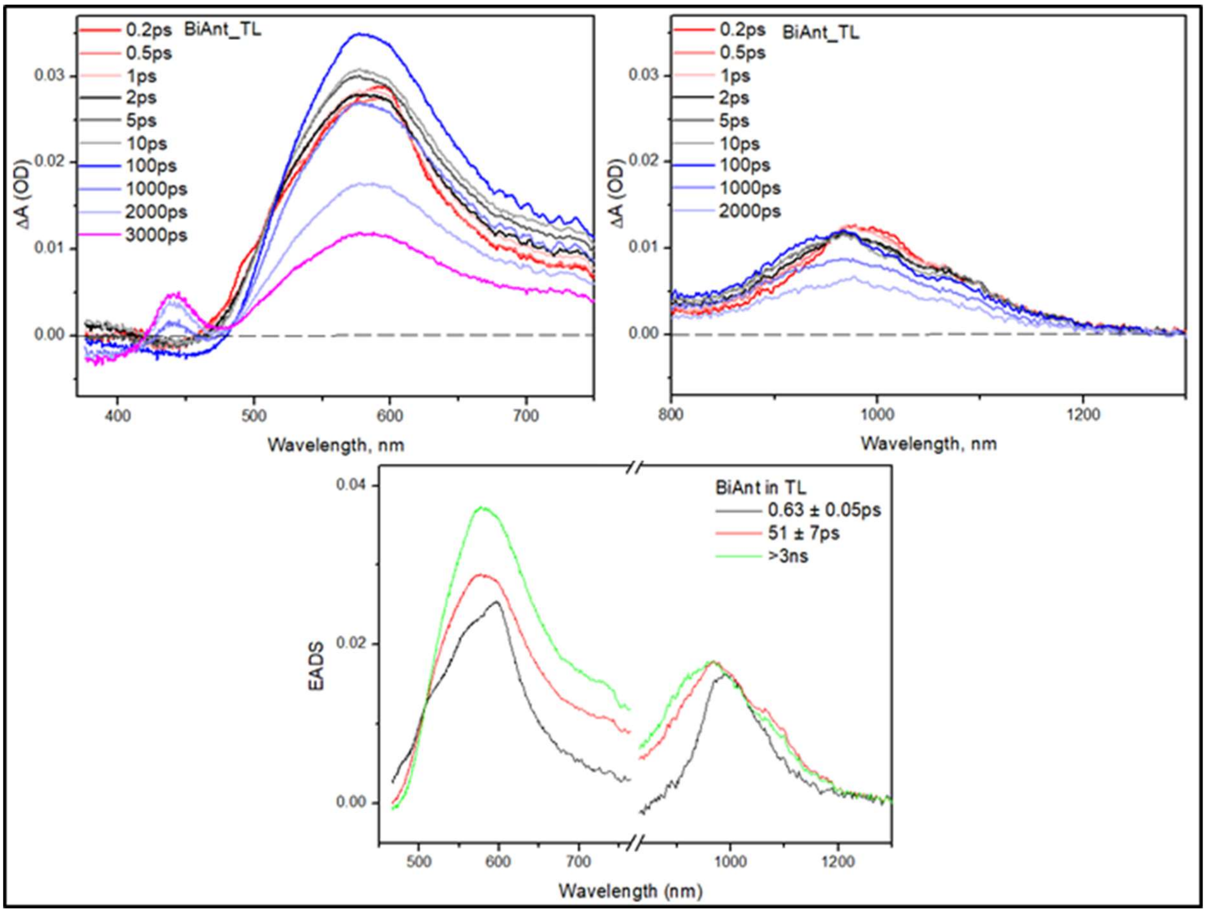

Figure S6. Transient absorption spectra and the evolution associated difference spectra (EADS) of 1,9' BA in toluene

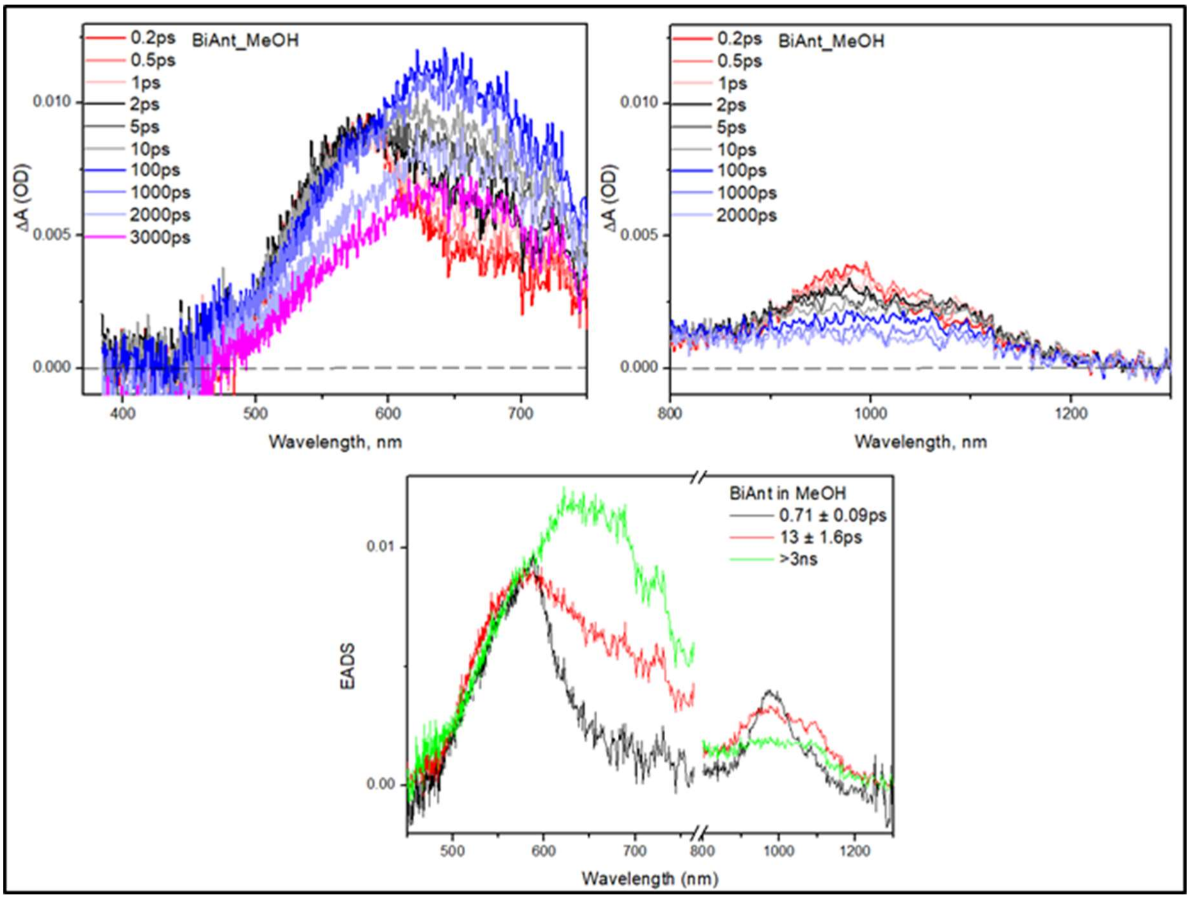

Figure S7. Transient absorption spectra and the evolution associated difference spectra (EADS) of 1,9’ BA in methanol

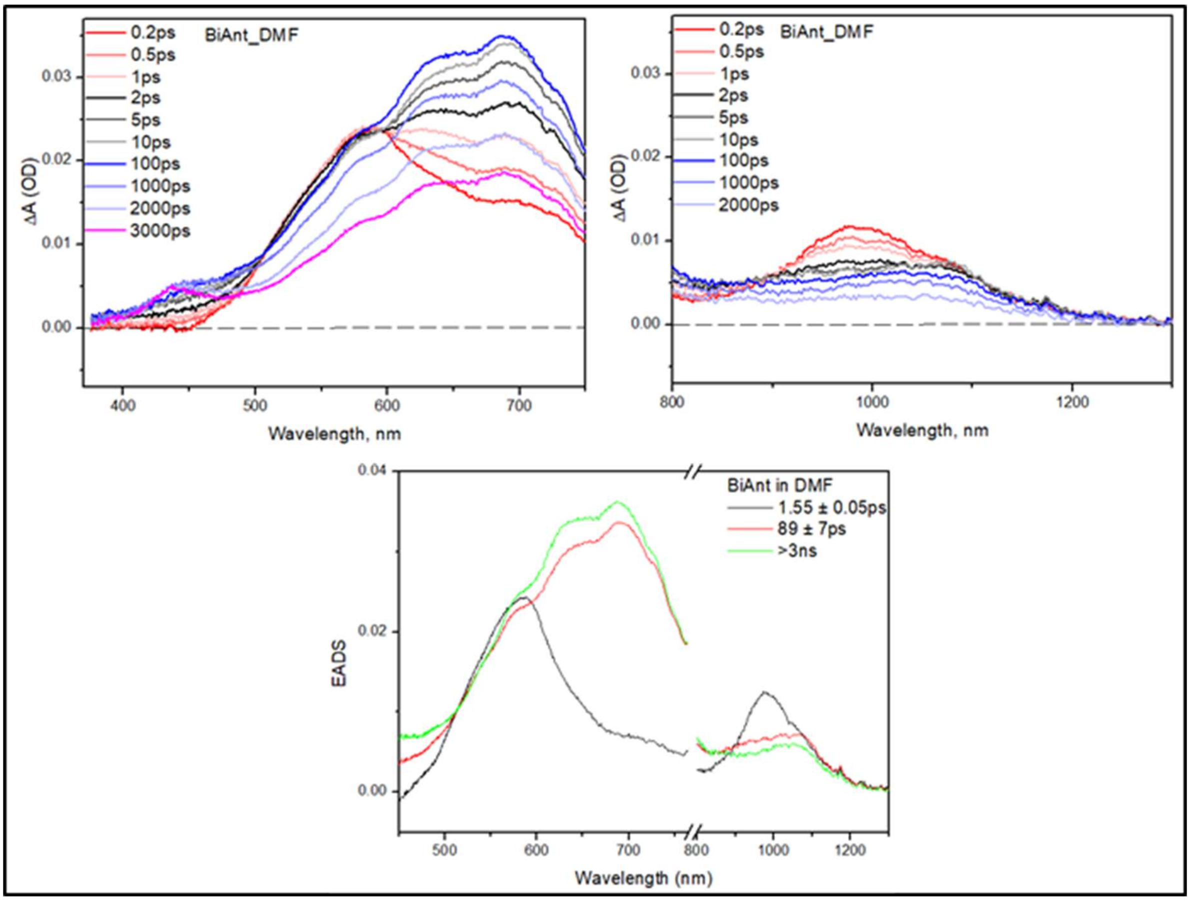

Figure S8. Transient absorption spectra and the evolution associated difference spectra (EADS) of 1,9’ BA in DMF

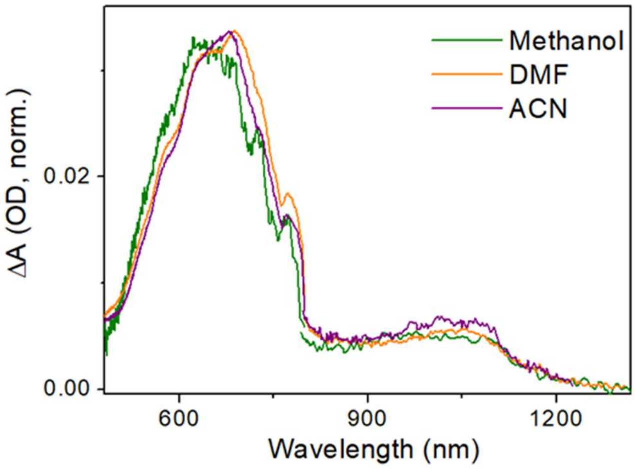

Figure S9. TA data of 1,9’BA corresponding to long-lived component in different solvents

Solvent	T /K	η /cP	T/η /K cP^{-1}	τ_1 /ps	τ_2 /ps	τ_F /ps	k_1 /ps^{-1}	k_2 /ps^{-1}
Pentane	295	0.24	1229.16667	0.2 ± 0.05	18 ± 4	4000	5	0.0553
Tol	295	0.59	500	0.63 ± 0.05	51 ± 7	4000	1.587	0.01936
CHX	295	1.02	289.21569	1.1 ± 0.2	91 ± 5	4000	0.909	0.01074
Tetradec	295	2.2	134.09091	5.5 ± 0.6	332 ± 20	4000	0.182	0.00276

Table S4. Table for rotational diffusion analysis of 1,9'BA in nonpolar solvents

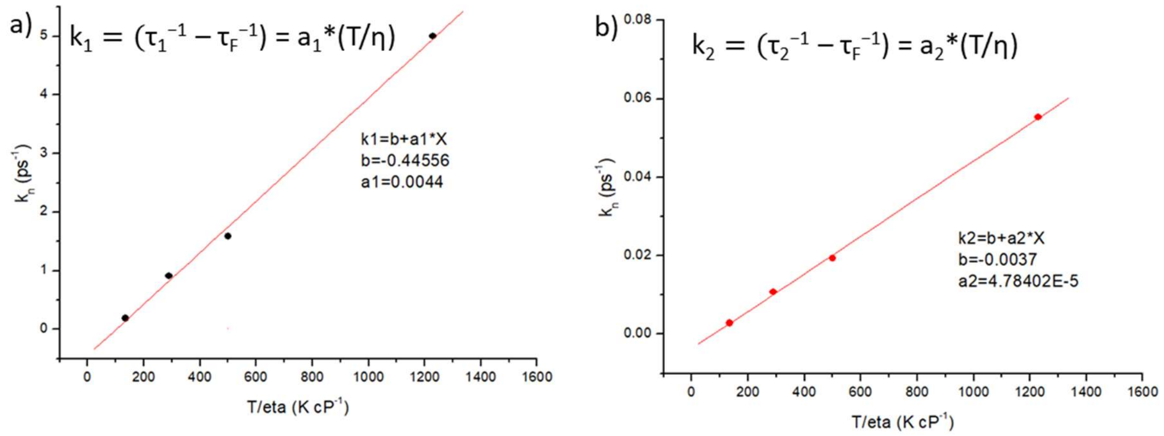

Figure S10. Rotational diffusion analysis of 1,9'BA in nonpolar solvents for the a) fast and b) slow relaxation times

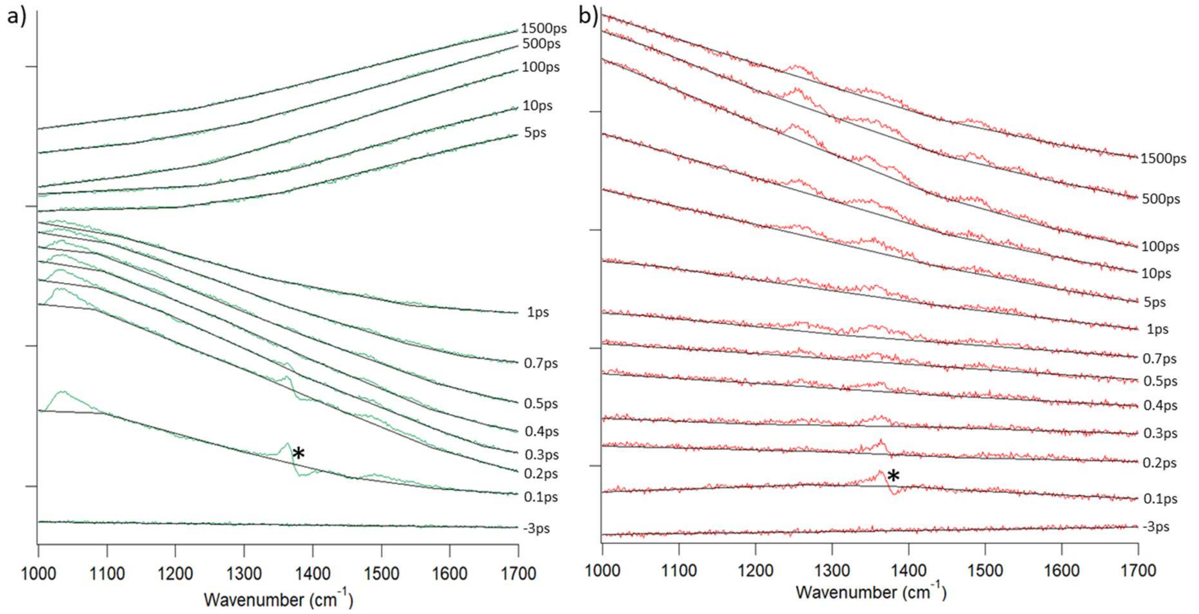

Figure S11. Raw excited state Raman data (green and red spectra for Raman pump at a) 560 and b) 650 nm respectively) of 1,9'BA in ACN at different time delays with the corresponding baselines shown (black lines). Asterisk (*) shows the solvent artifacts.

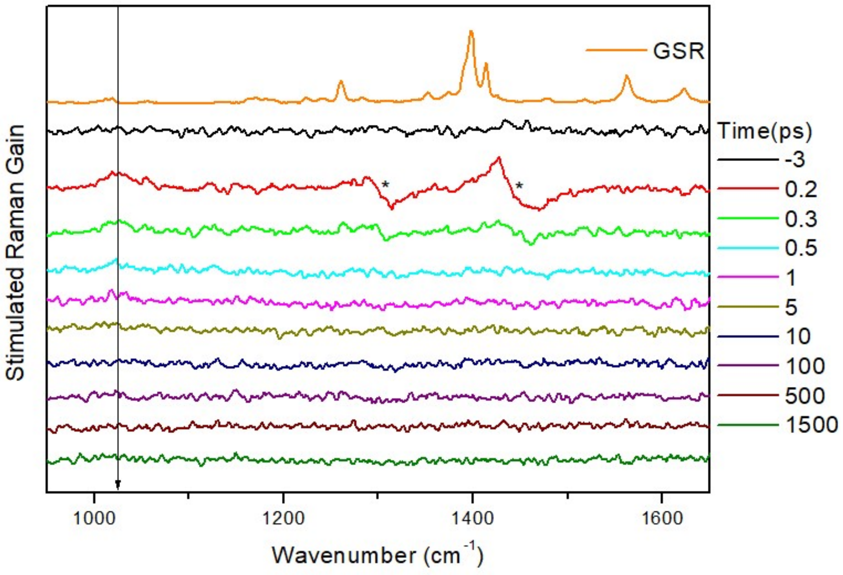

Figure S12. FSRS spectra of 1,9'BA in tetradecane at different time delays for Raman pump at 650 nm. GSR represents ground state spontaneous Raman and asterisk (*) shows the solvent artifacts.

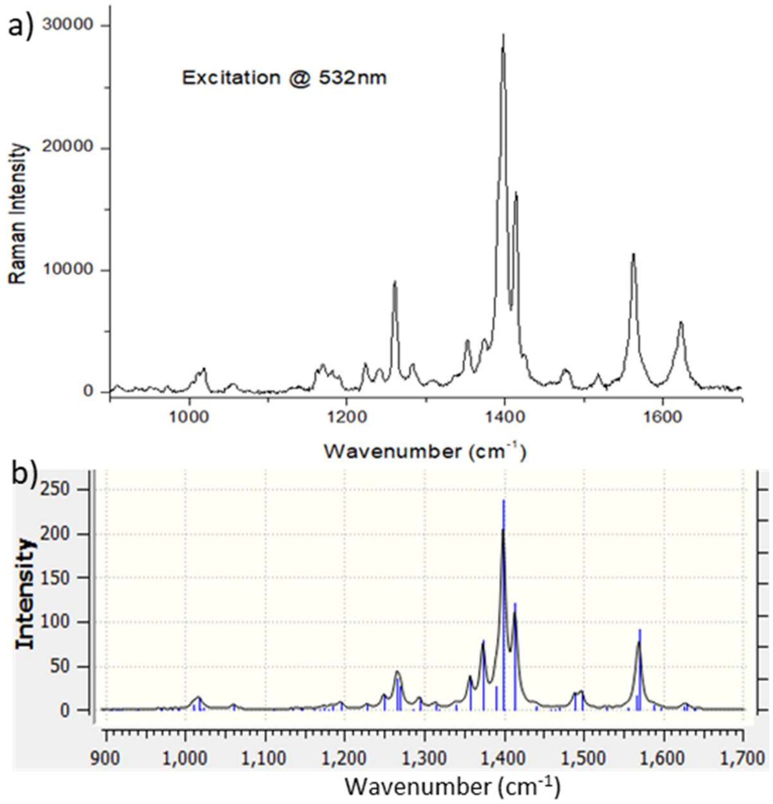

Figure S13. a) Spontaneous Raman recorded with 532 nm Laser on solid 1,9'BA. b) DFT Calculated Raman spectra using Gaussian 16. The basis set used was B3LYP-6311g (2d,2p).

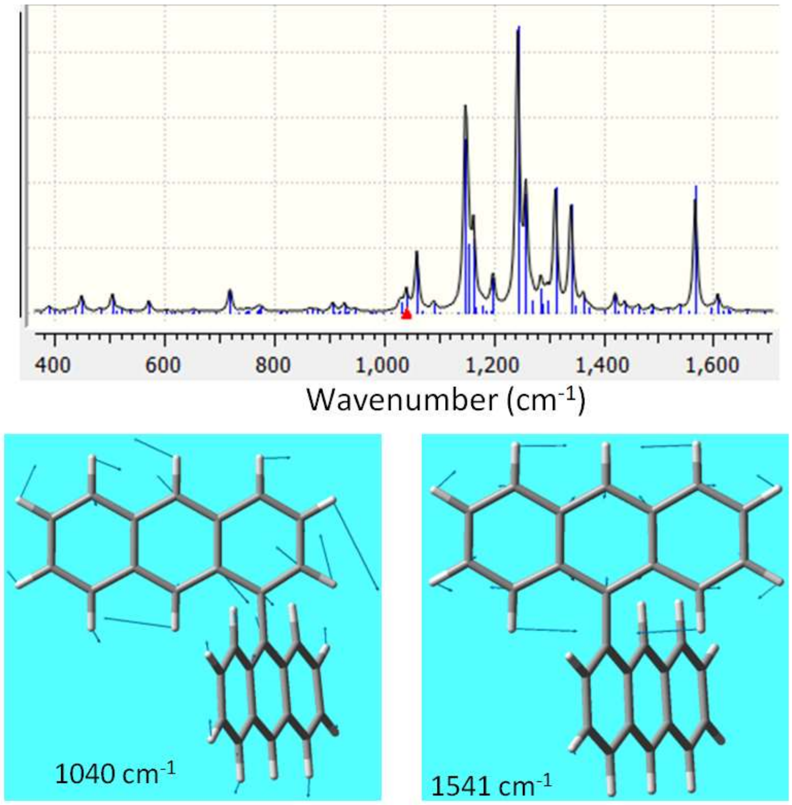

Figure S14. TDDFT Calculated S_1 Raman spectra (basis set: CAM-B3LYP 631g (d,p) and CPCM model with cyclohexane) of 1,9'BA in its optimized geometry (i.e. relaxed S_1). The modes at 1040 and 1541 cm^{-1} are shown.

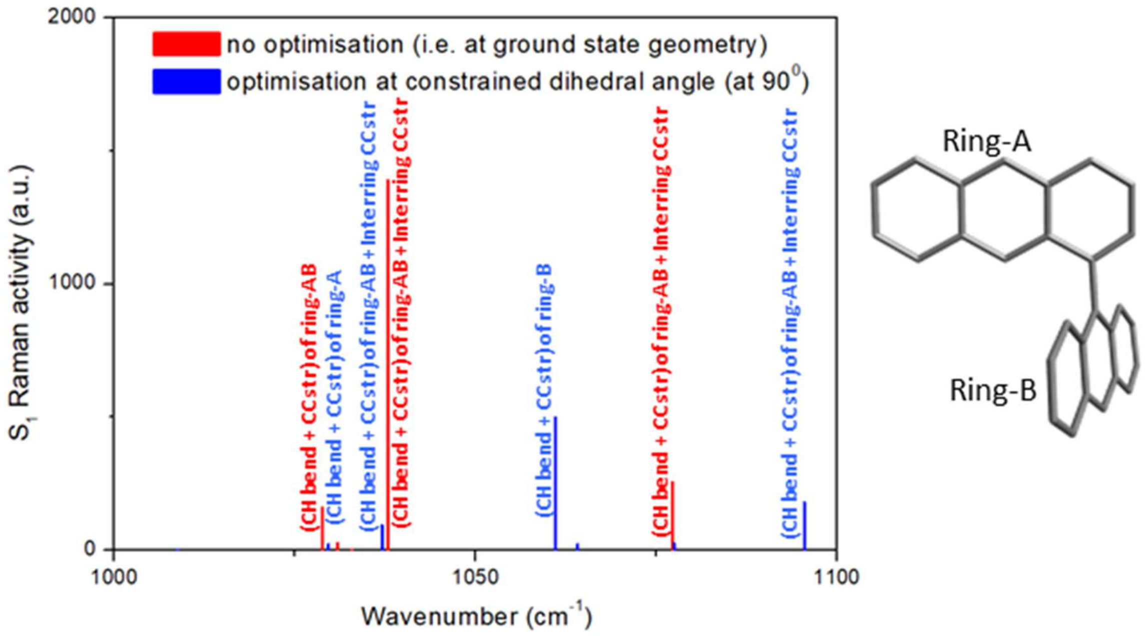

Figure S15. TDDFT Calculated S1 Raman spectra of 1,9'BA (basis set: CAM-B3LYP 631g (d,p) and CPCM model with cyclohexane) on the ground state geometry (no geometry optimization in S_1) and the optimized geometry with constrained dihedral angle (at 90°). The corresponding assignments of the modes are shown by red and blue texts respectively. Raman spectra are plotted for the 1000-1100 cm^{-1} region. 'str' represents C-C stretching frequency.

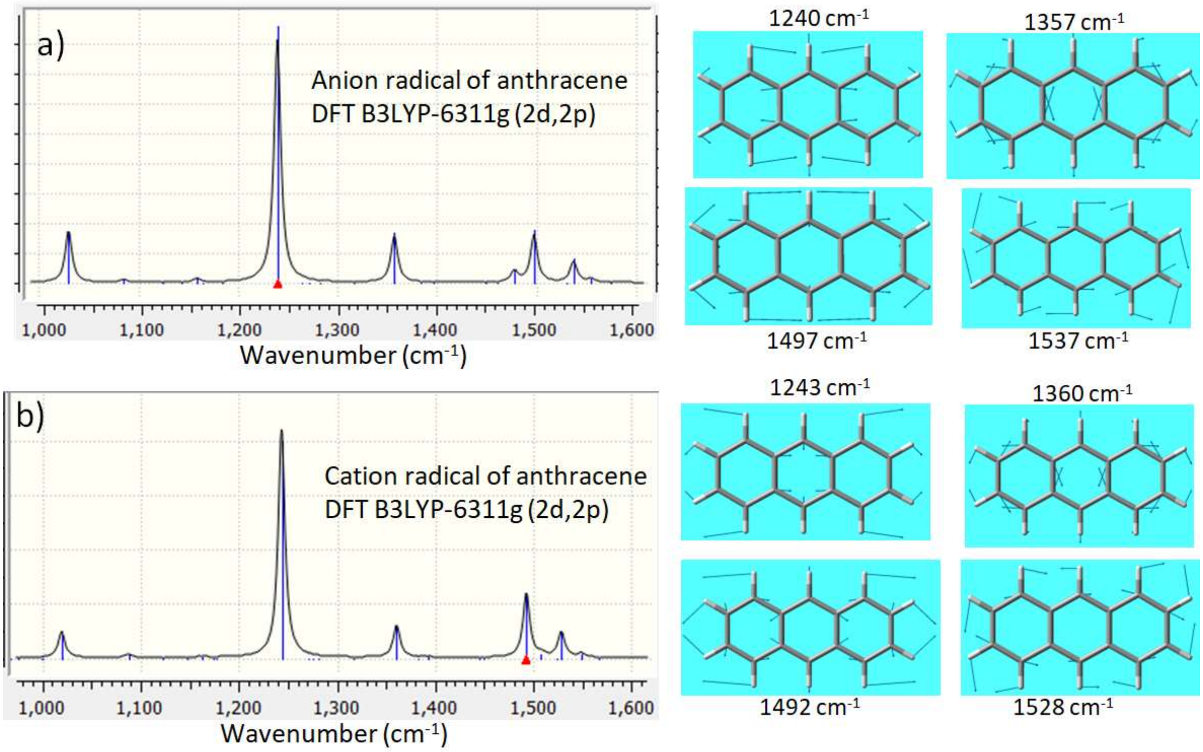

Figure S16. DFT Calculated Raman spectra (basis set: B3LYP-6311g (2d,2p) and CPCM model with acetonitrile) of a) anion radical and b) cation radical of anthracene. Some of the relevant frequencies are shown.

Atomic coordinates of 1,9'BA optimized at DFT B3LYP-6311g (2d,2p)/CPCM level of calculation:

C	0.31824100	-0.00001200	2.76549200
C	0.15758700	-0.00000600	1.40518200
C	-1.17230500	-0.00000400	0.85230200
C	-2.30047800	-0.00000600	1.75210100
C	-2.06511100	-0.00001000	3.15821500
C	-0.79434000	-0.00001400	3.64905400
C	-1.41377500	-0.00000100	-0.52381400
C	-3.59448400	-0.00000400	1.22753700
C	-3.83234900	-0.00000100	-0.14733100
C	-2.70872300	0.00000000	-1.04698900
C	-2.95903300	0.00000200	-2.45173100
H	-2.11380700	0.00000300	-3.12782200
C	-4.23421700	0.00000300	-2.93616900
C	-5.34452100	0.00000200	-2.04622400
C	-5.14930900	0.00000100	-0.69623100
H	-0.57638200	0.00000000	-1.20778900
H	1.31785400	-0.00001400	3.17913300
H	-2.91626000	-0.00001200	3.82673400
H	-0.61990600	-0.00001800	4.71643700
H	-4.43649300	-0.00000500	1.90906700
H	-4.40996900	0.00000400	-4.00337600
H	-6.34788400	0.00000300	-2.45014600
H	-5.99331100	0.00000000	-0.01842900
C	3.11413900	3.64547200	-0.76403000
C	3.66211300	2.46068600	-1.15560500
C	3.09617500	1.21598800	-0.74648700
C	1.92776400	1.22149400	0.09806600
C	1.38952800	2.48904600	0.48178800
C	1.96038800	3.65637000	0.06578800
C	3.64790100	0.00000800	-1.14476600
C	1.35920800	-0.00000100	0.51227900
C	1.92777000	-1.22149200	0.09806000
C	3.09618200	-1.21597700	-0.74649300

C	3.66212500	-2.46067000	-1.15561600
H	4.54080000	-2.44207000	-1.78755400
C	3.11415600	-3.64546000	-0.76404800
C	1.96040400	-3.65636800	0.06576800
C	1.38953800	-2.48904800	0.48177400
H	4.52606800	0.00001200	-1.77880200
H	3.55273700	4.58172600	-1.08156900
H	4.54078800	2.44209300	-1.78754400
H	0.51443500	2.51333800	1.11404200
H	1.53333000	4.60280000	0.36899400
H	3.55275800	-4.58171100	-1.08159100
H	1.53334800	-4.60280100	0.36896800
H	0.51444400	-2.51334700	1.11402500

Atomic coordinates of 1,9'BA optimized at TDDFT CAM-B3LYP-631g (d,p)/CPCM level of calculation:

C	0.51845100	-2.08819800	-1.70628800
C	0.25527300	-1.03766900	-0.84784800
C	-1.12308700	-0.79096000	-0.46396400
C	-2.16554300	-1.58429300	-1.05793900
C	-1.82057100	-2.61355300	-1.98036000
C	-0.51178100	-2.86618200	-2.28025000
C	-1.47190800	0.14584400	0.50536000
C	-3.49468800	-1.35302600	-0.69723100
C	-3.84227300	-0.38916400	0.24947400
C	-2.80179900	0.37105500	0.87502100
C	-3.15760200	1.33895000	1.86158500
H	-2.36616800	1.91145700	2.33578400
C	-4.46258600	1.54433800	2.19944900

C	-5.49725000	0.79171900	1.57406800
C	-5.19722200	-0.14508700	0.63085300
H	-0.69181800	0.72022700	0.99311400
H	1.54643600	-2.28677700	-1.99007000
H	-2.61674900	-3.20007600	-2.42815100
H	-0.25183800	-3.65359700	-2.97970200
H	-4.27622400	-1.95238200	-1.15613300
H	-4.72184700	2.28453700	2.94909700
H	-6.52952800	0.96976300	1.85673000
H	-5.98280200	-0.72320900	0.15378600
C	4.53401500	-1.92850100	1.93440900
C	4.56030800	-0.57481200	1.58512400
C	3.52619100	0.00924700	0.83669400
C	2.42371000	-0.80674300	0.40035800
C	2.40763100	-2.14715900	0.80786400
C	3.45761300	-2.70913500	1.55118700
C	3.53334900	1.38304200	0.52641900
C	1.36305900	-0.20348400	-0.36291300
C	1.41983300	1.19653200	-0.71027100
C	2.51656700	1.99421500	-0.23203900
C	2.57213100	3.35875600	-0.56398500
H	3.39955800	3.95161700	-0.18605000
C	1.60168100	3.94975100	-1.37519300
C	0.57142500	3.17354600	-1.88062400

C	0.49248600	1.81058000	-1.56017600
H	4.36312600	1.99219800	0.87489900
H	5.34827100	-2.35433500	2.51067100
H	5.38996700	0.05056900	1.90068600
H	1.56059100	-2.77033700	0.55067200
H	3.41066100	-3.75655900	1.82871500
H	1.66618400	5.00556800	-1.61533500
H	-0.17939900	3.61137200	-2.52952000
H	-0.31289700	1.22232100	-1.98189600

Characterization of 1,9'BA through NMR:

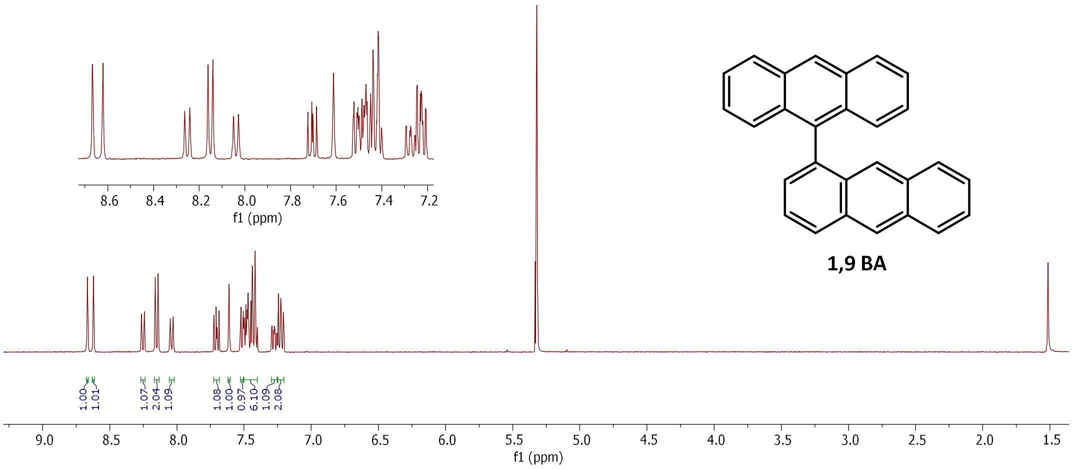

Figure S17. ^{1}H NMR Spectrum of compound 1,9'BA in CD_2Cl_2.

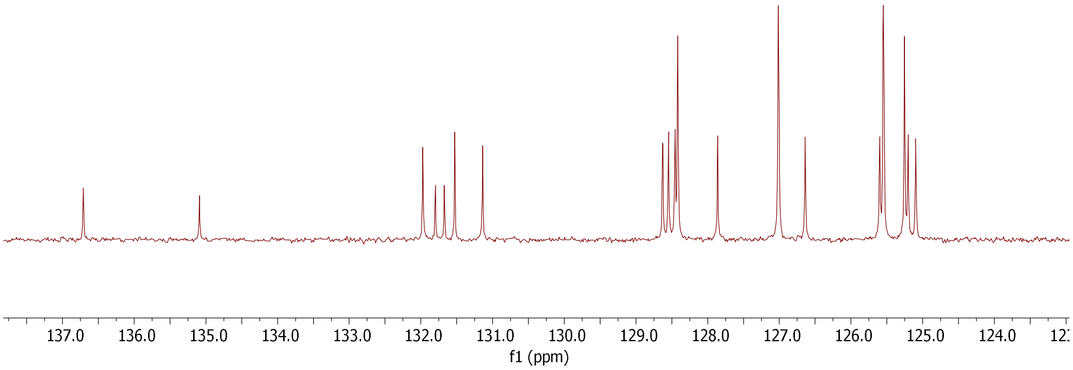

Figure S18. ^{13}C NMR Spectrum of compound 1,9' BA in CD_2Cl_2.

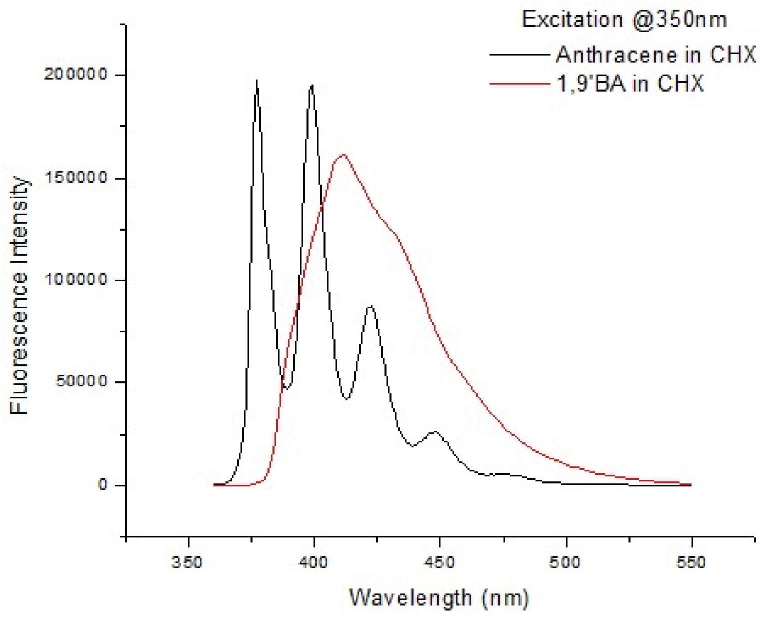

Figure S19: Emission spectra of anthracene and 1,9’BA for the measurement of fluorescence quantum yield.

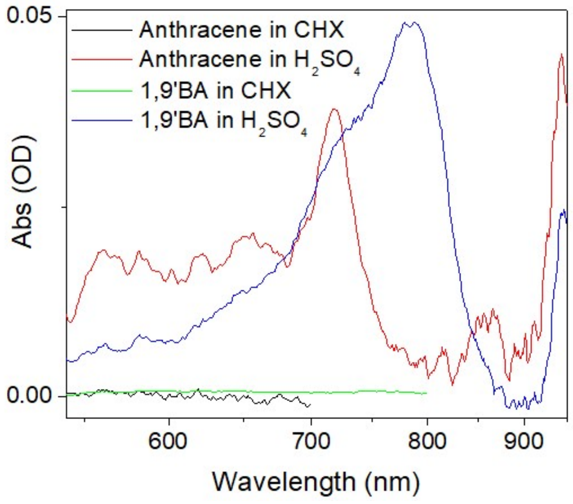

Figure S20: Absorption spectra of anthracene and 1,9’BA in cyclohexane and concentrated H_2SO_4 respectively.

References

(1) Roy, P.; Bressan, G.; Gretton, J.; Cammidge, A. N.; Meech, S. R. Ultrafast Excimer Formation and Solvent Controlled Symmetry Breaking Charge Separation in the Excitonically Coupled Subphthalocyanine Dimer. *Angewandte Chemie International Edition* **2021**, *60* (19), 10568-10572. DOI: 10.1002/anie.202101572.
(2) Snellenburg, J. J.; Laptenok, S. P.; Seger, R.; Mullen, K. M.; van Stokkum, I. H. M. Glotaran: A Java-Based Graphical User Interface for the R Package TIMP. *Journal of Statistical Software* **2012**, *49* (3), 1-22.
(3) Hall, C. R.; Conyard, J.; Heisler, I. A.; Jones, G.; Frost, J.; Browne, W. R.; Feringa, B. L.; Meech, S. R. Ultrafast Dynamics in Light-Driven Molecular Rotary Motors Probed by Femtosecond Stimulated Raman Spectroscopy. *J. Am. Chem. Soc.* **2017**, *139* (21), 7408-7414. DOI: 10.1021/jacs.7b03599.
(4) M. J. Frisch, G. W. T., H. B. Schlegel, G. E. Scuseria, M. A. Robb, J. R. Cheeseman, J. A. Montgomery, Jr., T. Vreven, K. N. Kudin, J. C. Burant, J. M. Millam, S. S. Iyengar, J. Tomasi, V. Barone, B. Mennucci, M. Cossi, G. Scalmani, N. Rega, G. A. Petersson, H. Nakatsuji, M. Hada, M. Ehara, K. Toyota, R. Fukuda, J. Hasegawa, M. Ishida, T. Nakajima, Y. Honda, O. Kitao, H. Nakai, M. Klene, X. Li, J. E. Knox, H. P. Hratchian, J. B. Cross, C. Adamo, J. Jaramillo, R. Gomperts, R. E. Stratmann, O. Yazyev, A. J. Austin, R. Cammi, C. Pomelli, J. W. Ochterski, P. Y. Ayala, K. Morokuma, G. A. Voth, P. Salvador, J. J. Dannenberg, V. G. Zakrzewski, S. Dapprich, A. D. Daniels, M. C. Strain, O. Farkas, D. K. Malick, A. D. Rabuck, K. Raghavachari, J. B. Foresman, J. V. Ortiz, Q. Cui, A. G. Baboul, S. Clifford, J. Cioslowski, B. B. Stefanov, G. Liu, A. Liashenko, P. Piskorz, I. Komaromi, R. L. Martin, D. J. Fox, T. Keith, M. A. Al-Laham, C. Y. Peng, A. Nanayakkara, M. Challacombe, P. M. W. Gill, B. Johnson, W. Chen, M. W. Wong, C. Gonzalez, and J. A. Pople. *Gaussian 03, Revision A.1,Gaussian, Inc., Pittsburgh PA,* **2003**.
