# Supplementary material for: Solvent Tuning Excited State Structural Dynamics in a Novel Bianthryl
Source: J Phys Chem Lett. 2023 Jan 3;14(1):253–9. doi: 10.1021/acs.jpclett.2c03469 (PMC9841557; doi:10.1021/acs.jpclett.2c03469)
Supplement: Supplementary file 2 — jz2c03469_si_002.pdf [file jz2c03469_si_002.pdf]

jz-2022-034695.R1

Name: Peer Review Information for "Solvent Tuning Excited State Structural Dynamics in a Novel Bianthryl"

First Round of Reviewer Comments

Reviewer: 1

Comments to the Author

In the manuscript titled ‘Solvent Tuning Excited State Structural Dynamics in a Novel Bianthryl’, the authors have reported a novel anthracene dimer exhibiting SBCS. The authors have employed transient absorption and stimulated Raman spectroscopy to understand the structural dynamics in different solvents. In non-polar solvent, the bianthryl system is found to undergo structural relaxation in the excited state to a polar emissive state. The results in the manuscript are interesting and the manuscript is worth publishing in JPC Letters after considering the suggestions.

1. The authors are recommended to cite recent review articles on SB-CS to provide a comprehensive outlook (Acc. Chem. Res. 2020, 53, 9, 1957–1968; Acc. Chem. Res. 2022, 55, 11, 1561–1572; ACS Energy Lett. 2022, 7, 2, 696–711).

2. No uncertainties/error bars are presented throughout the manuscript. The authors should give these values for all kinetic data.

3. Spectroelectrochemical experiments or chemically oxidized or reduced data are needed to verify the ion signal.

4. The authors are recommended to calculate the fluorescence quantum yield of the bianthryl system and include supporting information for better understanding.

Author's Response to Peer Review Comments:

We are grateful to the reviewer and editor for the positive assessments and helpful comments to improve the manuscript. We have now refined the main manuscript and SI based on reviewer and editor comments.

In the following reviewer and editor comments are normal typeface and shown in blue, our responses are black italic and new text is shown in red (as it also appears in the revised manuscript for reviewer convenience).

REVIEWER AND EDITOR COMMENTS

Reviewer 1:

Recommendation: This paper is publishable subject to minor revisions noted. Further review is not needed.

Comments: In the manuscript titled 'Solvent Tuning Excited State Structural Dynamics in a Novel Bianthryl', the authors have reported a novel anthracene dimer exhibiting SBCS. The authors have employed transient absorption and stimulated Raman spectroscopy to understand the structural dynamics in different solvents. In non-polar solvent, the bianthryl system is found to undergo structural relaxation in the excited state to a polar emissive state. The results in the manuscript are interesting and the manuscript is worth publishing in JPC Letters after considering the suggestions.

Reply: *Thank you. We believe the additions suggested (below) materially improved the manuscript.*

1. The authors are recommended to cite recent review articles on SB-CS to provide a comprehensive outlook (Acc. Chem. Res. 2020, 53, 9, 1957–1968; –1572; ACS Energy Lett. 2022, 7, 2, 696–711).

Reply: *We have added to the second lines of the introduction where SBCS is introduced:*

, a topic which continues to excite wide interest.[1-3]

1. Young, R. M.; Wasielewski, M. R., Mixed Electronic States in Molecular Dimers: Connecting Singlet Fission, Excimer Formation, and Symmetry-Breaking Charge Transfer. *Accounts of Chemical Research* **2020,** *53* (9), 1957-1968.
2. Estergreen, L.; Mencke, A. R.; Cotton, D. E.; Korovina, N. V.; Michl, J.; Roberts, S. T.; Thompson, M. E.; Bradforth, S. E., Controlling Symmetry Breaking Charge Transfer in BODIPY Pairs. *Accounts of Chemical Research* **2022,** *55* (11), 1561-1572.
3. Sebastian, E.; Hariharan, M., Symmetry-Breaking Charge Separation in Molecular Constructs for Efficient Light Energy Conversion. *ACS Energy Letters* **2022,** *7* (2), 696-711.

2. No uncertainties/error bars are presented throughout the manuscript. The authors should give these values for all kinetic data.

Reply: *We thank the reviewer for pointing out this omission. We have now added uncertainties for the global fit lifetime values in the main manuscript on Figure 2 and in Supplementary Figures S4-S8, and Supplementary Tables S3- S4.*

3. Spectroelectrochemical experiments or chemically oxidized or reduced data are needed to verify the ion signal.

Reply: *We have analysed both the anthracene and 1,9'BA in concentrated H_2SO_4 and added the following in the SI Figure S20 (below) which is referred to in the text as follows:*

confirmed for the electronic ground state in Figure S20

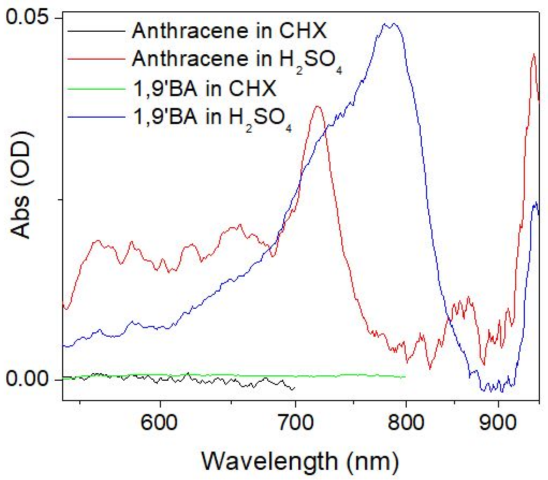

Figure S20. Absorption spectra of anthracene and 1,9'BA in cyclohexane and concentrated H_2SO_4 respectively.

4. The authors are recommended to calculate the fluorescence quantum yield of the bianthryl system and include supporting information for better understanding.

Reply: *We have now calculated the fluorescence quantum yield and added the following in the main text (p4)*

(the fluorescence quantum yield of 1,9'BA in cyclohexane was measured as 0.54 by a relative method, SI)

And added details in the SI as follows:

Emission quantum yield measurements: *The emission quantum yield (Q) of the 1,9'BA in CHX was determined using anthracene monomer as reference standard with a quantum yield of 0.36.(REF: Berlman, 1971) Concentrations of the samples were kept around 0.065 OD (at 350 nm) in a 1 cm cell. The emission spectra for sample and reference were collected at 350 nm excitation (SI Figure S19) keeping all the parameters (data accumulation time, slit width) the same. Yields were calculated according to:*

$$Q_f = Q_r.\left(\frac{I_f}{I_r}\right).\left(\frac{A_r}{A_f}\right).\left(\frac{n_f^2}{n_r^2}\right)$$

Where Q_f = fluorescence quantum yield of 1,9'BA; Q_r= quantum yield of Anthracene reference; A= absorbance (at 350 nm); I = integrated emission area; n = refractive index. The recovered quantum yield was 0.54.

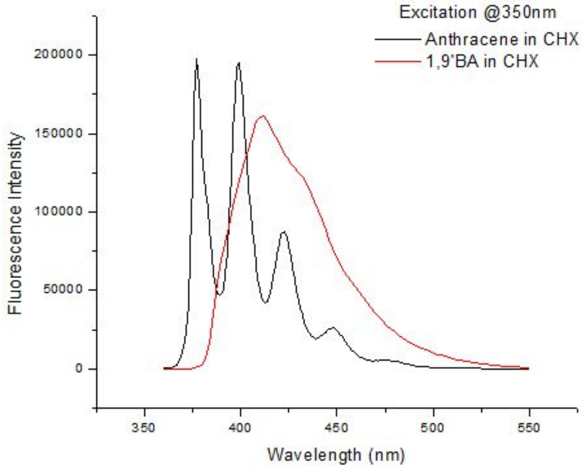

Figure 19: Emission spectra of anthracene and 1,9'BA for the measurement of fluorescence quantum yield.

Editor:

1) TOC Graphic: Provide a TOC image per journal guidelines (2 in x 2 in; on the same page as the abstract) with the heading "TOC Graphic" above the graphic.

Reply: *Done*

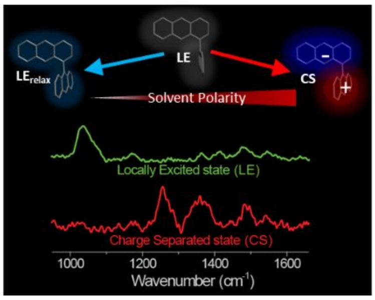

2) Abstract: Shorten the abstract to 150 words or fewer.

Reply: Done

3) Title: In both the main manuscript file and the Supporting Information, set the title in title case, with the first letter of each principal word capitalized.

Reply: Done

4) References: In both the main file and the supporting information, fix the style of all references to use JPCL formatting (check all references carefully). ***JPC Letters reference formatting requires

that journal references should contain: () around numbers, author names, article title (titles entirely in title case or entirely in lower case), abbreviated journal title (italicized), year (bolded), volume (italicized), and pages (first-last). Book references should contain author names, book title (in the same pattern), publisher, city, and year. Websites must include date of access.

Reply: The references have been updated using the JPCL style

5) Supporting Information Statement: The supporting-information (SI) paragraph in the main file is incomplete. Please list each item (including graphics) that can be found in the SI-for-Publication file individually.

Reply: The text now reads:

Supplementary Information Supplementary information describes the details of the experimental methods, additional figures and tables specifically: Experimental details on absorption, fluorescence, transient absorption and femtosecond stimulated Raman; details of computation of excited and ground state structures by DFT and TDDFT; data analysis and global fitting; temperature dependent emission spectra; tables of measured spectral transition wavelengths as a function of solvent; table and figure of fluorescence lifetimes; figures and analysis of solvent dependent transient absorption; table of data for rotational diffusion analysis and associated graphs; details of treatment of femtosecond stimulated Raman data; details of DFT and TDDFT calculations of Raman spectra; atomic coordinated from DFT and TDDFT calculations; NMR spectra of the synthesised 1,9BA.

6) Supporting Information: Please number SI pages in the following format: “S1, S2…”

Reply: *Done*
